# Arms race between anti‐silencing and RdDM in noncoding regions of transposable elements

**DOI:** 10.15252/embr.202256678

**Published:** 2023-06-05

**Authors:** Taku Sasaki, Kae Kato, Aoi Hosaka, Yu Fu, Atsushi Toyoda, Asao Fujiyama, Yoshiaki Tarutani, Tetsuji Kakutani

**Affiliations:** ^1^ The University of Tokyo Tokyo Japan; ^2^ National Institute of Genetics Mishima Japan; ^3^ The Graduate University for Advanced Studies (SOKENDAI) Mishima Japan; ^4^ Present address: Nihon BioData Corporation Kawasaki Japan; ^5^ Present address: Kihara Institute for Biological Research Yokohama City University Yokohama Japan

**Keywords:** anti‐silencing, DNA methylation, RdDM, transposable elements, Chromatin, Transcription & Genomics, Plant Biology

## Abstract

Transposable elements (TEs) are among the most dynamic parts of genomes. Since TEs are potentially deleterious, eukaryotes silence them through epigenetic mechanisms such as repressive histone modifications and DNA methylation. We previously reported that *Arabidopsis* TEs, called *VANDAL*s, counteract epigenetic silencing through a group of sequence‐specific anti‐silencing proteins, VANCs. VANC proteins bind to noncoding regions of specific *VANDAL* copies and induce loss of silent chromatin marks. The VANC‐target regions form tandem repeats, which diverge rapidly. Sequence‐specific anti‐silencing allows these TEs to proliferate with minimum host damage. Here, we show that RNA‐directed DNA methylation (RdDM) efficiently targets noncoding regions of *VANDAL* TEs to silence them *de novo*. Thus, escape from RdDM could be a primary event leading to the rapid evolution and diversification of sequence‐specific anti‐silencing systems. We propose that this selfish behavior of TEs paradoxically could make them diverse and less harmful to the host.

## Graphical abstract

Analyses of VANC anti‐silencing systems of *VANDAL* transposable elements in *Arabidopsis* reveal that VANC and RdDM compete with each other. Escaping from RdDM could be a selective force inducing the diversification of the anti‐silencing systems.

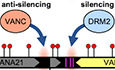

## Introduction

Transposable elements (TEs) are major constituents of the large genomes of vertebrates and plants. As the movement of TEs is mutagenic and potentially deleterious to the host, most of TEs are silenced by epigenetic mechanisms, such as DNA methylation, histone modifications, and RNAi (Hosaka & Kakutani, 2018). On the contrary, at least some TEs have been occasionally active on an evolutionary timescale, as they are very dynamic evolutionarily. Interestingly, population epigenomic analyses in the flowering plant *Arabidopsis thaliana* revealed variation in the epigenetic states of TEs among natural accessions (Kawakatsu *et al*, 2016; Quadrana *et al*, 2016), consistent with the view that TEs are occasionally activated in natural populations. Thus, an important question would be how TEs are activated and silenced in natural populations.

*Arabidopsis* serves as an ideal model organism to investigate the regulation of TEs, where precise TE sequences throughout the genome and trans‐acting mutations affecting epigenetic modifications of TEs are available (Ito & Kakutani, 2014; Quadrana *et al*, 2016). In *Arabidopsis* mutants defective in DNA methylation, diverse TEs are transcriptionally derepressed and mobilized, demonstrating the importance of DNA methylation for the immobilization of these *Arabidopsis* TEs (Miura *et al*, 2001; Singer *et al*, 2001; Kato *et al*, 2003; Lippman *et al*, 2004; Mirouze *et al*, 2009; Tsukahara *et al*, 2009). Some TEs are mobilized# in the background of loss‐of‐function mutants of *MET1* (*METHYLTRANSFERASE 1*), a Dnmt1 class DNA methyltransferase maintaining cytosine methylation in symmetric CpG context (hereafter referred to as mCG). In addition, cytosine methylation in non‐CpG context (hereafter mCH) is catalyzed by CMT2 (CHROMOMETHYLASE 2) and CMT3 in *Arabidopsis*. While some TEs, such as *EVADE*, are mobilized^#^ when mCG is compromised, other TEs, such as *CACTA1* or *AtGP3*, are mobilized^#^ only when both mCG and mCH are lost (Kato *et al*, 2003; Mirouze *et al*, 2009; Tsukahara *et al*, 2009). These TEs are also mobilized^#^ in mutants defective in chromatin remodeling factor DDM1 (decrease in DNA methylation 1), which is necessary for both mCG and mCH in heterochromatic TEs (Vongs *et al*, 1993; Jeddeloh *et al*, 1999; Zemach *et al*, 2013).

Interestingly, diverse TEs activated by the *ddm1* mutation, such as *CACTA1* and *VANDAL21*, remain mobile even in the wild‐type (WT) background (Kato *et al*, 2004; Fu *et al*, 2013; Quadrana *et al*, 2019). The inheritance of TE mobility correlated with the inheritance of DNA methylation status (Vongs *et al*, 1993; Kakutani *et al*, 1999; Kato *et al*, 2004). Nevertheless, recovery of DNA methylation is found in TEs with small RNAs that target them (Teixeira *et al*, 2009). In plants, small RNA is involved in the *de novo* DNA methylation pathway called RdDM (RNA‐directed DNA methylation). Components of RdDM have been extensively studied in *Arabidopsis* (Matzke & Mosher, 2013; Cuerda‐Gil & Slotkin, 2016; Fultz & Slotkin, 2017; Sigman *et al*, 2021). Multiple pathways triggered by small RNA converge to *de novo* DNA methylation by DRM1 (DOMAINS REARRANGED METHYLASE 1) and DRM2. Nonautonomous TE copies often induce the silencing of autonomous copies (Slotkin *et al*, 2005; Burgess *et al*, 2020; Sasaki *et al*, 2022a), and it is assumed that this homology‐based silencing is mediated by small RNA. However, evidence for the impact of RdDM on TE mobility in natural populations remains limited. More importantly, it remains largely unknown how heritable DNA methylation and silencing of TEs can be erased during evolution.

As a mechanism for the activation of TEs, we have previously shown that TEs named *VANDAL*s can counteract epigenetic silencing. *VANDAL21* is one of the TEs silenced in WT plants, but it is mobilized when DNA methylation is lost (Tsukahara *et al*, 2009). An intriguing feature of *VANDAL21* and other *VANDAL* family TEs is that they encode proteins named VANCs, which have anti‐silencing activity (Fu *et al*, 2013; Hosaka *et al*, 2017a; Sasaki *et al*, 2022a); expression of a VANC protein from a transgene induces loss of DNA methylation, transcriptional derepression of the encoded genes, and mobilization of some of them (Fu *et al*, 2013; Hosaka *et al*, 2017a; Sasaki *et al*, 2022a). Thus, the anti‐silencing activity of VANC proteins counteracts the genome defense by the host to silence TEs by DNA methylation.

Interestingly, the anti‐silencing effects of VANC proteins were very sequence specific; expression of VANC21 protein induces loss of DNA methylation only in *VANDAL21* copies, while other sequences, including other *VANDAL* member copies, are unaffected (Fu *et al*, 2013). ChIP‐seq analyses revealed that VANC21 protein is localized specifically in noncoding regions of *VANDAL21* copies, which are enriched in tandem repeats with specific short motifs (Hosaka *et al*, 2017a). That short motif is also important for binding of VANC21 to DNA *in vitro*. A related *VANDAL* TE, called *VANDAL6*, encodes related proteins, and the expression of one protein, named VANC6, induces loss of DNA methylation in *VANDAL6* copies and related TE copies but not *VANDAL21* copies. The regions affected by VANC6 also have tandem repeats with motifs different from those in VANC21 targets. Thus, although *VANDAL21* and *VANDAL6* are similar in their overall sequences, the encoded VANC proteins and their target sequences are differentiated and have distinct specificities.

Antidefense/anti‐RNAi mechanisms are widespread in viruses, but the target specificity is generally low in these pathways; and such nonspecific antidefense often reduces host fitness severely (Bivalkar‐Mehla *et al*, 2011; Zhao *et al*, 2016). While global anti‐silencing mechanisms are also known in TEs (Nosaka *et al*, 2012; Cosby *et al*, 2019), it is less common than that in viruses. That may be because horizontal transfer is less efficient in TEs than in viruses and severe reduction of host fitness by global antidefense would be deleterious for the survival of that TE within the host population. In contrast to the global anti‐silencing, the sequence‐specific anti‐silencing by VANCs would allow the *VANDAL* TEs to proliferate with minimum damage to the host. This strategy would be advantageous for the survival of the *VANDAL* TEs within the host population, especially in the long term (Hosaka *et al*, 2017a; Sasaki *et al*, 2022a).

Here, we genetically characterized the ability of the host to re‐silence the activated *VANDAL21* TEs. Genetic analyses revealed that RdDM targets DNA methylation in noncoding regions of activated TEs to immobilize them. Thus, both silencing and anti‐silencing target noncoding regions of TEs. Furthermore, as small RNAs can function *in trans* to silence TEs, the rapid evolution of VANC targets could also be understood as an escape from RdDM.

## Results

### The *VANDAL21* transgene activates endogenous *VANDAL21* , but the activated endogenous copy is re‐silenced when the transgene is segregated away

**Figure 1:**
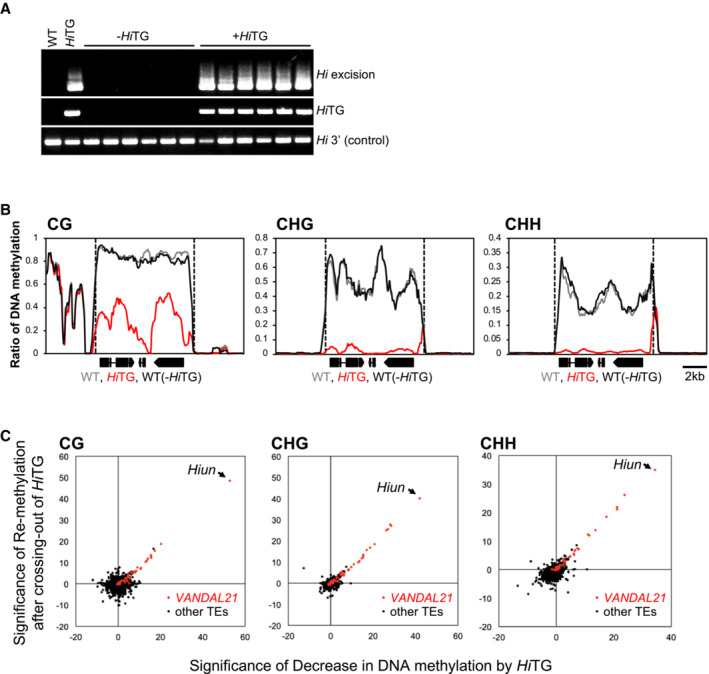
Endogenous *VANDLA21* TEs are re‐silenced when *Hi*TG is segregated away Excision of endogenous *Hi* in plants with and without *Hi*TG obtained from the same parental plant.Patterns of DNA methylation for each cytosine context in an endogenous *Hi* locus calculated from WGBS data. Vertical dashed lines indicate the termini of *Hiun*.Comparison of the significance of changes in DNA methylation by *Hi*TG (*x*‐axis) and those when *Hi*TG was segregated (*y*‐axis) for all *Arabidopsis* TEs (*n* = 31,189). The significance of changes in DNA methylation was assessed as described in Fu *et al* (2013) (for details, see Materials and Methods). Dots in red and black indicate *VANDAL21* and other TEs, respectively. Source data are available online for this figure.

As is the case for most TEs in plant genomes, *VANDAL* family TEs are generally heavily methylated and silenced in WT plants. However, when WT plants are transformed with a full‐length endogenous mobile copy of *VANDAL21*, called *Hiun* (*Hi*), endogenous *Hi* loses DNA methylation, is transcriptionally derepressed, and is mobilized (Fu *et al*, 2013; Fig 1 and Appendix Fig S1). Interestingly, when the *Hi* transgene (*Hi*TG) was segregated in the self‐pollinated progeny of the transgenic line, endogenous *Hi* was immobilized immediately (Fu *et al*, 2013; Fig 1A), and the DNA methylation recovered almost to the original WT level (Fu *et al*, 2013; Fig 1B). Analogous loss and recovery of DNA methylation can also be seen in other copies of *VANDAL21* (Fig 1C). These results suggest that hosts have efficient mechanisms to methylate and silence *VANDAL21* in the absence of VANC21 protein.

### RdDM contributes to the re‐silencing of *Hi*

**Figure 2:**
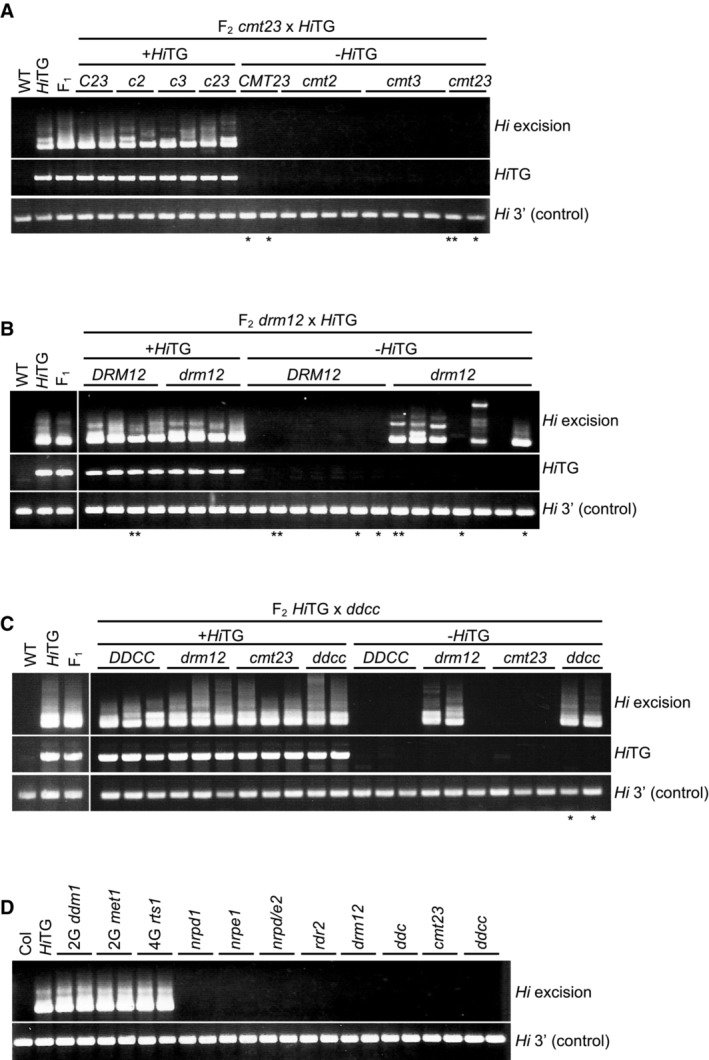
Re‐silencing of *VANDAL21* depends on RdDM A–CExcision of endogenous *Hi* in *cmt23* (A), *drm12* (B), and *ddcc* (C) mutant backgrounds when *Hi*TG is segregated away. Plants with * and ** were used for WGBS, and the results of those with ** were used for the analysis shown in figures.DExcision of endogenous *Hi* in mutant backgrounds of epigenetic regulators. *drm12*: *drm1 drm2* double mutant, *ddc*: *drm1 drm2 cmt3* triple mutant, *cmt23*: *cmt2 cmt3* double mutant, and *ddcc*: *drm1 drm2 cmt2 cmt3* quadruple mutant. Histone acetyltransferase HDA6/RTS1 is reported as important for silencing of TEs, and TEs activated in *hda6* mutant are shared with those activated in *met1* (To *et al*, 2011). Source data are available online for this figure.

To understand the underlying mechanism for this re‐silencing, we examined the factors necessary for re‐silencing. The *Arabidopsis* genome encodes three types of DNA methyltransferases for DNA methylation in CpG and non‐CpG contexts (hereafter called mCG and mCH, respectively). MET1 maintains mCG, CMT2, and CMT3 direct mCH to regions with histone H3 lysine 9 methylation (H3K9me), and DRMs catalyze both the mCG and mCH *de novo* RdDM pathways (Lloyd & Lister, 2022). In the *cmt2 cmt3* double mutant (hereafter called *cmt23*), *Hi* was re‐silenced when *Hi*TG segregated away (Fig 2A). On the contrary, in the background of the *drm1 drm2* double mutant (hereafter called *drm12*), *Hi* often remained mobile even after the loss of *Hi*TG (Fig 2B). Mutations in the other components of the RdDM pathway, such as *NRPD1*, *NRPE2*, *NRPE1*, and *RDR2*, also compromised the re‐silencing (Appendix Fig S2). Taken together, these results demonstrate that RdDM is effective for re‐silencing *VANDAL21*. In fact, small RNA is accumulating in *VANDAL* TEs in WT (Appendix Fig S3).

Although *Hi* remained mobile when RdDM was compromised, we could not detect mobilization in a few sibling plants (two *drm12* plants shown in Fig 2A; two *nrpd1*, one *nrpe1*, and two *nrpe2* plants shown in Appendix Fig S2) in F2, as well as their self‐pollinated F3 plants (Appendix Fig S4), suggesting that additional mechanisms may also contribute to the immobilization of *Hi*.

A possible backup for RdDM in regard to mCH is CMTs; mCH is lost almost completely in the quadruple mutant *drm1 drm2 cmt2 cmt3* (hereafter called *ddcc*; Stroud *et al*, 2014a). Then, we analyzed the immobilization of endogenous *Hi* in the *ddcc* mutant background. In the *ddcc* background, *Hi* remained mobile in all of the F2, F3, and F4 plants examined (Fig 2C and Appendix Fig S5), suggesting that CMTs are involved in the immobilization of *Hi* in the *drm12* mutant background. Importantly, without exposure to *Hi*TG, *ddcc* mutation does not induce the mobilization of endogenous *Hi* (Fig 2D), and small RNA is still accumulated in *VANDAL* TEs (Appendix Fig S3). Thus, *ddcc* mutation has a strong impact on the *de novo* immobilization of *Hi*, even though immobilized *Hi* remains immobile in the *ddcc* background.

The results above suggest that loss of mCH by *ddcc* does not affect the maintenance of immobilization. In striking contrast, *Hi* is mobilized when mCG is lost in the *met1* single mutant even without previous exposure to *Hi*TG, demonstrating the critical role of mCG for the maintenance of *Hi* immobilization (Fig 2D). Similarly, *Hi* was also mobilized in *ddm1* and *rts1* mutants, which also loses mCG in TEs (Fig 2D). Taken together, these results suggest that mCG has a strong impact on the maintenance of immobilization of *VANDAL21*, while mCH machinery is necessary for *de novo* silencing of *VANDAL21*.

### RdDM is necessary for *de novo* CG methylation in targets of VANC21

**Figure 3:**
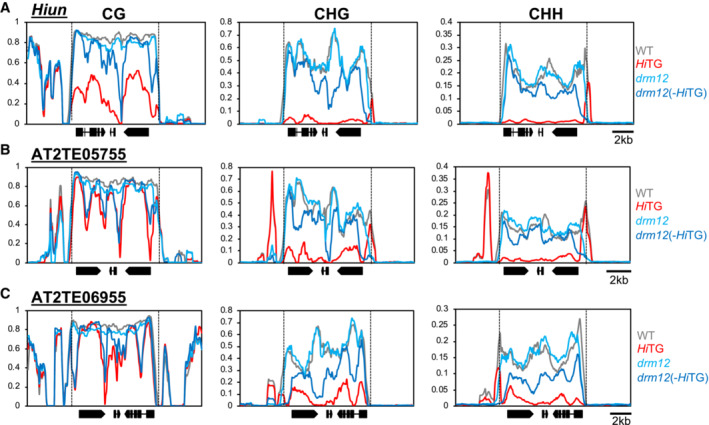
RdDM induces mCG in noncoding regions of *VANDAL21* TEs when *Hi*TG is segregated away A–CPatterns of DNA methylation in endogenous *Hi* (A), AT2TE05755 (B), and AT2TE06955 (C) in the *drm12* mutant background. “(−*Hi*TG)” indicates that *Hi*TG was segregated. Vertical dashed lines indicate the termini of TEs. Source data are available online for this figure.

**Figure 4:**
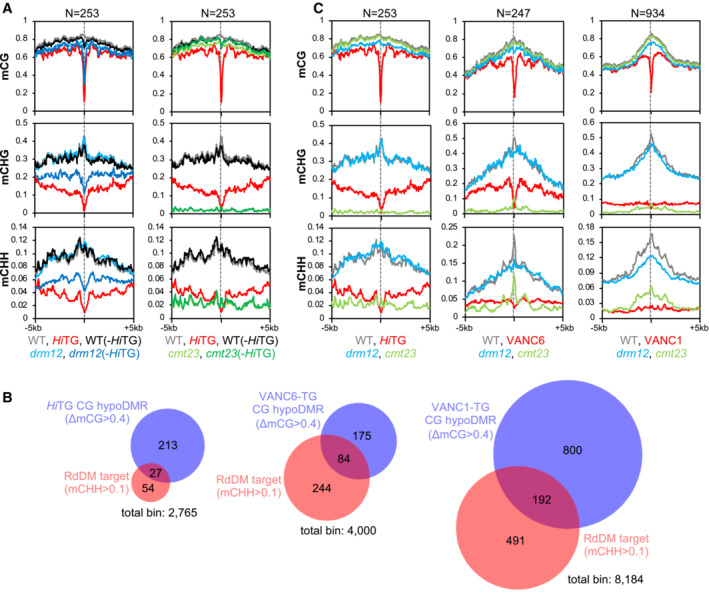
Target regions of RdDM and VANCs overlap Patterns of DNA methylation in flanking regions of VANC21 targets when *Hi*TG was segregated in *drm12* (left) and *cmt23* (right) mutant background. Gray dashed lines indicate the points of VANC targets. The same analysis using VANC21‐binding regions detected by ChIP‐seq as VANC21 target regions is shown in Appendix Fig S6.Venn diagrams showing the overlap between targets of CG demethylation by VANCs and CHH methylation by RdDM. RdDM target regions are 3.13‐, 3.96‐, and 2.47‐fold overrepresented in VANC21, VANC6, and VANC1 target regions, respectively (for VANC21, VANC6, and VANC1, hypergeometric *P* = 1.88e‐08, 5.42e‐32, and 1.13e‐32, respectively).Patterns of DNA methylation around VANC21 (left), VANC6 (middle), and VANC1 (right) targets. Source data are available online for this figure.

The results above suggest the importance of mCG for controlling *VANDAL21*. In addition, the results demonstrate that RdDM is effective for *de novo* silencing of *VANDAL21* activated by VANC21. RdDM affects both mCG and mCH, and CH methylase CMTs can also contribute to *de novo* silencing. To further understand the role of mCG and mCH, we examined the impact of mutations in the RdDM machinery on the recovery of mCG and mCH. In the experiment for observing the recovery of DNA methylation after the loss of *Hi*TG, *drm12* mutations affected the DNA methylation recovery in noncoding regions more than in coding regions (Fig 3A). These trends were also found in *VANDAL21* copies other than *Hi* (Fig 3B and C). Notably, ChIP‐seq results suggest that VANC21 protein localizes in noncoding regions of *VANDAL21* copies (Hosaka *et al*, 2017a). Indeed, DRM activity affected the recovery of methylation around the peak of VANC21 target regions, which was defined by the loss of mCG (Fig 4A and Appendix Fig S6). This effect was more conspicuous in mCG than in mCH (Fig 4A). These results are consistent with the conclusion that mCG is important for *Hi* activity (Fig 2D) and strongly suggest that RdDM silences *Hi* through *de novo* CG methylation of the target of VANC21.

Complementary results were obtained for the effect of the *cmt23* mutations; in *cmt23*, mCG remained, although mCH was abolished nearly completely in coding regions (Appendix Fig S7A). Under such conditions, *Hi* was completely silenced and immobilized (Fig 2A), again suggesting that mCG, rather than mCH, is critical for the maintenance of *Hi* silencing. Such complementary effects of RdDM and CMTs were also seen in other *VANDAL21* copies (Appendix Fig S7B and C).

### Targets of RdDM and VANC overlap

The results above demonstrate that RdDM targets noncoding regions, as is the case for anti‐silencing by VANC21. We, therefore, compared mCG DMRs hypomethylated in the presence of VANC21 (ΔmCG > 0.4 in *Hi*TG) to RdDM targets (mCHH > 0.1 in *cmt23*). RdDM targets were 3.13‐fold overrepresented in VANC21‐targeted regions (mCG DMRs; hypergeometric *P*‐value = 1.88e‐08; Fig 4B and Appendix Fig S8). Similar features were also observed in VANC1 and VANC6. Within the VANC1 and VANC6 target regions, RdDM targets were 2.32‐ and 3.96‐fold overrepresented based on the same criterion (hypergeometric *P*‐value = 1.13e‐32 and 5.42e‐32, respectively, Fig 4B). Moreover, target regions of VANC1 and VANC6 showed strong peaks of mCH in *cmt23* mutants, which reflects the activity of RdDM, although such a peak was less obvious in target regions of VANC21 (Fig 4C). Taken together, these results indicate that RdDM and VANC regulate common target regions in opposite directions.

### Differential CG methylation between *Hi*TG and endogenous *Hi*

*Hi*TG induces reactivation of *VANDAL21*, which includes activation of the endogenous VANC21 gene (Appendix Fig S1). Thus, an interesting question is why the activated endogenous VANC21 gene can be the target of re‐silencing, while that of *Hi*TG can be expressed robustly. Endogenous *Hi* and *Hi*TG have the same sequences except for a few synonymous SNPs, but their responses to RdDM are different: while *Hi*TG activity is constant, endogenous *Hi* shows activity only when *Hi*TG is present, and *Hi* is targeted and silenced by RdDM when *Hi*TG is segregated away. The differences in sensitivity to RdDM may reflect differences in the DNA methylation patterns. Indeed, bisulfite sequencing using locus‐specific primers revealed that the DNA methylation status was different between endogenous *Hi* and *Hi*TG; while mCG remained in activated endogenous *Hi* (*Hiun* ^*Hi*TG^), *Hi*TG was free from mCG and mCH (Appendix Fig S9). Because their DNA sequences are nearly identical, differential mCG status is likely to be a determinant for sensitivity to RdDM.

**Figure 5:**
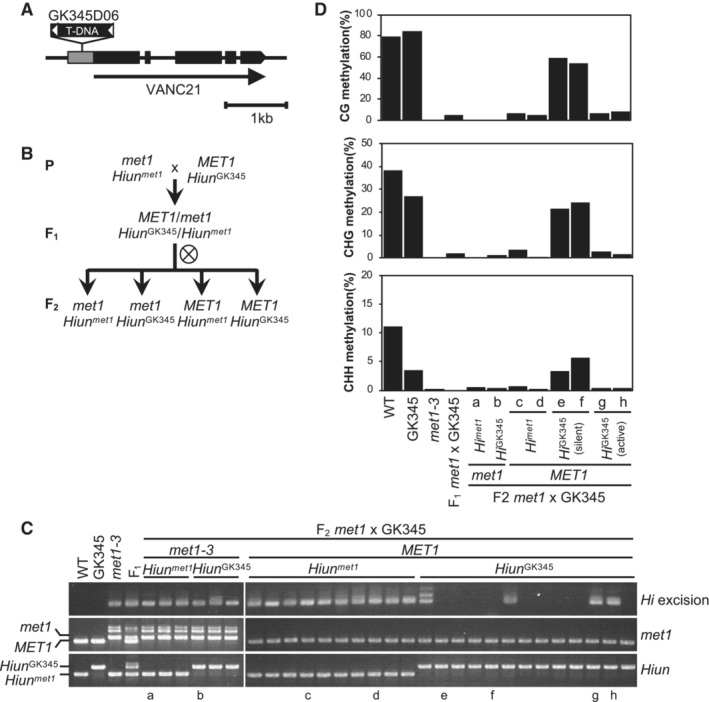
Loss of CG methylation makes *Hi* refractory to RdDM‐mediated silencing Schematic structure of GK345D06 (GK345), which has a T‐DNA insertion within the noncoding region of *Hi*.Genetic scheme to generate CG hypomethylated *Hi*. *met1* mutation induced a loss of CG methylation in *Hi* (*Hiun* ^*met1*^), and GK345, which has *Hi* with DNA methylation similar to WT (*Hiun* ^GK345^), enabled us to distinguish their origin.
*Hi* excision in F_2_ plants obtained from *met1* x GK345. Genotyping results of *MET1* and *Hi* loci are also shown. (a–h) indicate individuals who are analyzed for their DNA methylation status in (D).DNA methylation status in endogenous *Hi* calculated from the bisulfite sequencing results. Source data are available online for this figure.

To determine whether mCG is required for silencing of *Hi* by RdDM, we examined the behavior of *Hi* activated by the *met1* mutation. The *met1‐3* mutation induces a global loss of mCG and endogenous *Hi* is activated (Rigal *et al*, 2016, Fig 2D). We examined the behavior of *met1*‐activated *Hi* after introduction into the WT background. To distinguish between the origins of *Hi*, we utilized the GK‐345D05 line for the WT parent; this line has a T‐DNA insertion within the noncoding region of *Hi* (*Hiun* ^GK345^) (Fig 5A and Appendix Fig S10). We crossed the GK‐345D05 line with the *met1‐3* mutant, which has an endogenous *Hi* copy with mCG hypomethylation (*Hiun* ^*met1*^), and generated the F2 population by self‐pollination of F1 individuals (Fig 5B). In the segregating F2 generation, we analyzed the effects of *Hiun* ^*met1*^ and *Hiun* ^GK345^ on the excision of endogenous *Hi*. As endogenous *Hi* was always active in the *met1* mutant background, the effect of *Hiun* ^*met1*^ and *Hiun* ^GK345^ was monitored in the WT *MET1* background. Even in *MET1*, *Hiun* ^*met1*^ was actively excised, while most *Hiun* ^GK345^ was re‐silenced (Fig 5C). Consistent with their activity status, DNA methylation was restored in *Hiun* ^GK345^, while *Hiun* ^*met1*^ remained hypomethylated despite intact RdDM machinery (Fig 5D). These results indicate that complete loss of mCG makes *Hi* refractory to silencing by RdDM.

### CMT‐mediated body mCH represses the transcription of *VANDAL* ‐encoded genes

The results above suggest that mCG in noncoding regions of *Hi* is important for its activity. This is consistent with the view that DNA methylation around transcription start site (TSS) is critical for gene activity. Interestingly, however, VANCs have strong effects to reduce mCH in internal coding regions (gene bodies), which are apart from TSS, within the *VANDAL* copies (Fu *et al*, 2013; Hosaka *et al*, 2017a; Sasaki *et al*, 2022a). While mCH in noncoding regions is target of RdDM, gene body mCH is often catalyzed by CMTs (To *et al*, 2020; To & Kakutani, 2022).

**Figure 6:**
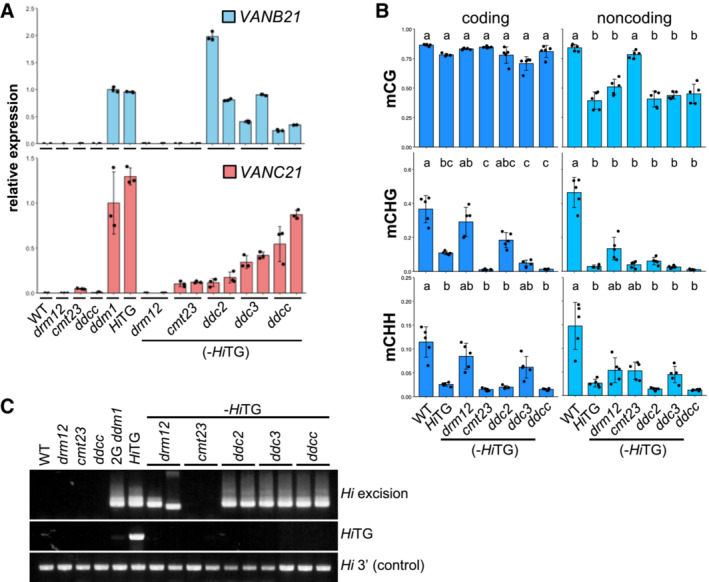
Gene body mCH induces transcriptional repression of *VANDAL*‐encoded genes Expression of *VANB21* (At2g23490) and *VANC21* (At2g23480) measured by RT–qPCR. The values were normalized to the expression levels in the *ddm1* mutant. For each genotype, the average values and standard deviations of the three technical replicates are shown. Note that we could not detect the expression of *VANC21* in *drm12* (−*Hi*TG), although endogenous *Hi* retained mobility (Fig 6C). This finding can likely be explained by the expression of *VANA21*, a transposase of *VANDAL21*, but we failed to compare the expression levels of this gene because of its very low expression level.DNA methylation status of coding and noncoding regions of five *VANDAL21* TEs (for detail, please see Appendix Fig S11B). The average values and standard deviations for each region are shown. One‐way ANOVA with Tukey's multiple comparisons test was used to determine the significant differences (*P* < 0.01).Excision of endogenous *Hi* detected by PCR. For RT–qPCR shown in (A) and excision analysis shown in (C), RNA and DNA, respectively, were extracted from the same plants. Source data are available online for this figure.

Interestingly, the mobility of *Hi* was more stably inherited in *ddcc* than in *drm12* (Fig 2 and Appendix Figs S4 and S5). The difference between *ddcc* and *drm12* is mainly in body mCH (Appendix Fig S7). Consistent with the view that body mCH induces transcriptional repression, RT–qPCR revealed that the transcription of genes encoded within *Hi* was much higher in *ddcc* (−*Hi*TG) than in *drm12* (−*Hi*TG) (Fig 6A). Partial loss of body mCH in *drm12 cmt3* (*ddc3*) and *drm12 cmt2* (*ddc2*) mutant background resulted in partial activation. CMT2 and CMT3 preferentially catalyze mCHH and mCHG, respectively (Fig 6B and Appendix Fig S11), as is the case in the other TEs (Zemach *et al*, 2013; Stroud *et al*, 2014a), and it is interesting that they do not seem to be redundant for silencing *Hi*. In addition, excision analysis of an endogenous *Hi* in the *ddc2* and *ddc3* mutant backgrounds also suggested that both CMT2 and CMT3 are required for effective immobilization of *Hi* (Fig 6C and Appendix Fig S12). While differential functions of mCHG and mCHH are still elusive, VANCs affect both of them (Fig 6B). Taken together, these results suggest that the VANC‐induced loss of mCH in coding regions also affects the activity of *Hi*.

## Discussion

We have previously shown that the anti‐silencing factor VANC21 binds to noncoding regions of *VANDAL21* TEs and induces loss of DNA methylation and mobilization (Hosaka *et al*, 2017a). In this report, we characterized the mechanisms by which the host re‐silences the activated *VANDAL21*: when the transgene expressing VANC21 is segregated, the RNAi‐based *de novo* DNA methylation mechanism RdDM efficiently reintroduces DNA methylation in noncoding regions of the affected *VANDAL21* TEs and immobilizes them.

The binding regions of VANC proteins have specific motif sequences in tandem repeat organization. The tandem repeats contract and expand frequently and evolve synchronously. We have previously proposed that the synchronous evolution of short target motifs enables each of the *VANDAL* families to have different sequence specificities and proliferate with minimum host damage (Hosaka *et al*, 2017a). On the contrary, tandem repeats can be sources of siRNA (Martienssen, 2003) and targets of RdDM (Alleman *et al*, 2006). We found an overlap between the targets of RdDM and targets of VANC1/VANC6, although the overlap was less obvious for the targets of VANC21 (Fig 4B and C). This unique feature of *VANDAL21* may reflect the fact that these elements are still proliferating and efficiently escaping RdDM. It is tempting to speculate that the short‐term advantage of differentiation of the VANC systems for the TEs is the escape from RdDM. For example, although *VANDAL21* and *VANDAL6* are similar in their overall sequences, they are different in sequences where VANC21 and VANC6 proteins affect (Hosaka *et al*, 2017a), presumably enabling escape of *VANDAL21* copies from RdDM targeting to *VANDAL6* copies. Such short‐term advantage of the diversification of the anti‐silencing systems as an escape from RdDM would lead to their long‐term advantage to maintain host fitness.

Compared with *met1* or *ddm1* mutant, which affect the maintenance of mCG, loss of RdDM function has only a minor impact on the TE derepression within the *Arabidopsis* genome (Huettel *et al*, 2006; Ito *et al*, 2011; Baduel *et al*, 2021). Consistent with this general trend, *Hi* is not activated in RdDM mutant, but RdDM has significant importance for *de novo* silencing of *Hi* (Fig 2 and Appendix Fig S2).

**Figure 7:**
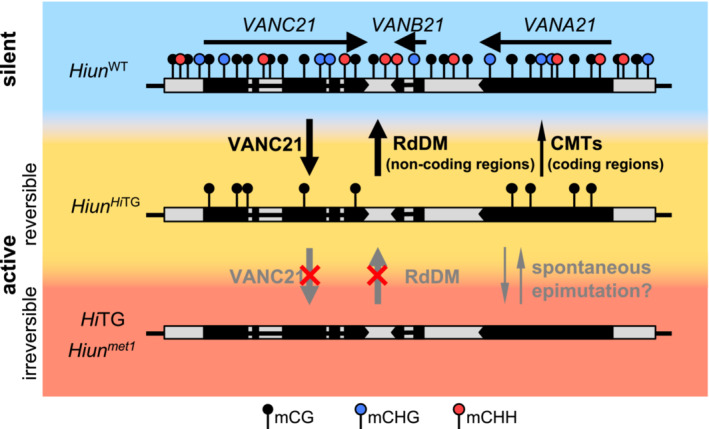
Possible model of the relationship between VANC21‐mediated anti‐silencing and RdDM There are three different epigenetic states of *Hi*: constantly active (irreversible), active but reversible, and silent. *Hi*TG and *Hiun* ^*met1*^ are constantly active, while endogenous *Hi* is active only when *Hi*TG co‐exists (*Hiun* ^*Hi*TG^) and reverts to a silent state (*Hiun* ^WT^) when *Hi*TG is segregated away. Among them, DNA methylation states are also different. *Hi*TG is free from DNA methylation, *Hiun* ^*Hi*TG^ has mCG, and the silent *Hiun* ^WT^ has both mCG and mCH. mCG has an important role in the recruitment of RdDM.

An intriguing feature of the effects of VANC on DNA methylation is that these effects spread outside the regions to which it binds and affects the entire TE. Although the effect is strongest in noncoding regions where VANC binds, surrounding coding regions also showed a significant loss of DNA methylation, especially in the CH context. Thus, anti‐silencing by VANC seems to have at least two layers of effects: local effects on mCG and mCH in noncoding regions where it binds and additional effects, mainly for mCH, in surrounding regions (Fig 7). While the biological effect of VANC in noncoding regions seems significant, the role and control of VANC for hypomethylation in coding regions remain enigmatic. We showed that gene body mCH contributes to the transcriptional repression of *VANB21* and *VANC21* (Fig 6). Thus, the spreading of mCH loss has a significant impact on the stable maintenance of the anti‐silencing effect. Interestingly, when the *VANC* transgene is segregated, loss of mCH in coding regions is recovered even in mutants of the RdDM machinery. Thus, in addition to the RdDM to silence *VANDAL*s, RdDM‐independent *de novo* silencing mechanisms may also function in coding regions. Consistent with this observation, RdDM‐independent recovery of mCH of TE genes is generally observed after the introduction of the WT gene to mutants defective in mCH and H3K9me2 (To *et al*, 2020). In addition, *VANDAL21* activated by *met1* mutation remains active even in the *MET1* WT background. Thus, complete loss of mCG generates an allele refractory to RdDM‐based silencing, perhaps through preventing the induction of DNA methylation or recruitment of RdDM machineries; the effect of mCG on *de novo* establishment of mCH has also been detected in other TE genes (Chan *et al*, 2006; Sasaki *et al*, 2014; To *et al*, 2022). In addition, other multiple positive feedback mechanisms involved in mCG are also suggested (Choi *et al*, 2021; To *et al*, 2022; To & Kakutani, 2022). Cross talk among multiple silencing pathways would achieve effective protection of the host genome from threat by TEs.

The loss of DNA methylation in TEs by the transposon‐encoded protein was also found in other TEs. Noticeable example is *Spm* originally found by McClintock (1951, 1958), which shows switching of activity during development. An *Spm*‐encoded protein called TnpA has been shown to activate *Spm*, which correlates with DNA demethylation (Schläppi *et al*, 1994; Cui & Fedoroff, 2002). Another example is MuDR in maize. MURA, a transposase encoded in autonomous MuDR, also induces DNA hypomethylation in TIRs of nonautonomous copies (Burgess *et al*, 2020). Importantly, DNA hypomethylation induced by MURA reverts default DNA methylated status when MuDR is lost (Lisch *et al*, 1995; Burgess *et al*, 2020). This relationship between MURA and nonautonomous copy resembles that between *Hi*TG and endogenous *Hi*. Thus, although responsible factors and underlying mechanisms are diverse, anti‐silencing by TEs may be more common than previously thought.

In this report, we showed that both VANCs and RdDM regulate noncoding regions of *VANDAL*s as common targets in opposite directions. Another unique feature of *VANDAL* TEs is degenerated TIRs (terminal inverted repeats; Le *et al*, 2000; Yu *et al*, 2000; Fu *et al*, 2013). The degeneration of TIRs can also be understood in terms of escape from RdDM; as long TIRs of *Mutator*‐like TEs are often the source of siRNA (Burgess *et al*, 2020; Sasaki *et al*, 2022a), degeneration of TIRs might compromise the pathway to induce *de novo* methylation of terminal regions of TEs because of low homology. Although a causative link remains to be tested, both degeneration of TIRs and the presence of VANC‐related genes are commonly seen in *VANDAL* TEs. The arms race between RdDM and VANCs on the regulation of transpositional activity likely resulted in highly specific and divergent anti‐silencing mechanisms of VANCs and contributed to the successful amplification of *VANDAL* TEs with minimal damage to the host.

## Materials and Methods

### Plant materials

Isolation of *met1‐3*, *ddm1‐1*, and *rts1‐1*/*hda6‐7* has been reported previously (Vongs *et al*, 1993; Aufsatz *et al*, 2002; Saze *et al*, 2003). The T‐DNA insertion mutants *drm1‐2* (SALK_031705), *drm2‐2* (SALK_150863), *cmt2‐3* (SALK_012874), *cmt3‐11 t* (SALK_148381), *rdr2‐2* (SALK_059661), *nrpd1‐3* (SALK_128428), *nrpe1‐11* (SALK_029919), *nrpd/e2‐2* (SALK_046208), and GK‐345D05 were obtained from the *Arabidopsis* Biological Resource Center (Alonso *et al*, 2001; Kleinboelting *et al*, 2012). A transgenic line containing the full‐length sequence of *Hi* (*Hi*TG) was reported by Fu *et al* (2013). All materials used in this research are Col background.

### Detection of *Hi* excision

The excision of endogenous *Hi* was detected by nested PCR as described in Fu *et al* (2013). Briefly, DNA was isolated from mature leaves using Nucleon PhytoPure (GE Healthcare), and 10 ng DNA was used for nested PCR as a template. The PCR conditions for the first round of amplification were 94°C for 1 min, followed by 25 cycles of 94°C for 15 s, 56°C for 30 s, and 72°C for 45 s, with a final extension at 72°C for 3 min. PCR products were diluted 20 times with water, and 1 μl was used for the second round of PCR. The PCR conditions were the same as those for the first PCR except that the cycle number was changed to 30 cycles. The primers used for nested PCR are listed in Appendix Table S1.

### DNA methylation analysis

For bisulfite sequencing, 300 ng of genomic DNA isolated with Nucleon PhytoPure (GE Healthcare) was digested by *Pst* I in a 20 μl reaction mix. Digested DNA was ethanol‐precipitated and eluted with 20 μl distilled water and then denatured by adding 2.2 μl 3 M NaOH and kept at 37°C for 30 min. Denatured DNA was treated with 208 μl urea/bisulfite solution (7.5 g urea and 7.6 g sodium bisulfite dissolved in 20 ml water, adjusted to pH 5.0) and 12 μl 10 mM hydroquinone followed by 30 cycles of 95°C for 30 s and 55°C for 15 min. Bisulfite‐treated DNA was purified by a GeneClean III Kit (MP Biomedicals), eluted with 20 μl distilled water, and then desulfonated by adding 2.2 μl 3 M NaOH and incubating at 37°C for 15 min. Finally, DNA was ethanol‐precipitated and eluted with 20 μl distilled water. Converted DNA was used as a template for PCR using EpiTaq HS (TaKaRa). The PCR conditions were 94°C for 1 min, followed by 35 cycles of 94°C for 15 s, 55°C for 30 s, and 72°C for 1 min, with a final extension at 72°C for 3 min. After gel electrophoresis, amplified DNA was purified and cloned into the pGEM T‐Easy Vector System (Promega). At least 14 copies were sequenced for each sample. The primers used for this experiment are shown in Appendix Table S1.

For WGBS analysis, DNA extraction, bisulfite treatment, and library preparation were performed as described previously (Fu *et al*, 2013), and sequencing (paired‐end, 150 bp) was performed by Macrogen Japan. Reads were trimmed using Trimmomatic (version 0.33) with the following parameters: “ILLUMINACLIP:TruSeq3‐PE.fa:2:30:10 LEADING:3 TRAILING:3 SLIDINGWINDOW:4:15 MINLEN:36” (Bolger *et al*, 2014). Trimmed reads were mapped to the *Arabidopsis* genome (TAIR10) by Bismark ver. 0.15.0 (Krueger & Andrews, 2011). The significance of changes in DNA methylation status was calculated as value (Mn/Cn − Mt/Ct)/(1√Cn + 1√Ct), where Mn, Cn, Mt, and Ct are methylated cytosine (M) and total cytosine (C) counts mapped for each TE in the nontransgenic (n) and transgenic (t) plants, respectively, as previously described (Fu *et al*, 2013). For the analysis of DNA methylation in *ddcc* mutant, publicly available WGBS data (GSE51304) were used (Stroud *et al*, 2014a; Data ref: Stroud *et al*, 2014b).

For the analysis of DNA methylation status shown in Fig 6B, we selected five full‐length *VANDAL21* TEs which have a complete set of coding genes (*VANA21*, *VANB21*, and *VANC21*), and ORFs of them were annotated using publicly available RNA‐seq data in *ddm1* mutant (GSE93584; Oberlin *et al*, 2017a; Data ref: Oberlin *et al*, 2017b). DNA methylation in coding (exons) and noncoding (intergenic regions, introns, and TIRs) regions were calculated. List of five *VANDAL21* TEs and their annotations is shown in Appendix Fig S11B.

### DMR detection

The DNA methylation ratio in each cytosine context within a 50 bp bin was calculated genome‐wide, and regions whose CG methylation ratio changed more than 0.4 within each VANC target (*VANDAL21* for VANC21, *VANDAL6*, *7*, *8*, *17*, and *AT9TSD1* for VANC6, and *VANDAL1*, *2*, *22*, *1 N1*, and *2 N1* for VANC1) were defined as DMRs. Because the DNA methylation status in TG is different from the endogenous status (Appendix Fig S9), bins overlapping with *AT2TE42810* (*VANDAL21*, *Hiun*), *AT4TE25050* (*VANDAL6*), and *AT1TE56425* (*VANDAL1*) were excluded. For DMR detection in VANC6‐TG and VANC1‐TG, publicly available WGBS data (DRA006000 and DRA013178, respectively) were used (Hosaka *et al*, 2017a; Data ref: Hosaka *et al*, 2017b; Sasaki *et al*, 2022a; Data ref: Sasaki *et al*, 2022b).

### Hypergeometric test

For each VANC‐target *VANDAL* family, TEs with > 2 CG hypoDMRs were selected as VANC‐target TEs. The DNA methylation ratio within a 50 bp bin for VANC‐target TEs was calculated in Col, transgenic plants, and *cmt23* mutants. As a control, regions with similar sizes were randomly selected using “shuffle” command of bedtools (Quinlan & Hall, 2010) and analyzed using the same criteria. Venn diagrams were made using BioVenn (Hulsen & de Vlieg, 2008).

### Expression analysis

Total RNA was isolated from mature leaves using TRIzol Reagent (Invitrogen) and treated with RQ1 RNase‐free DNase (Promega). cDNA was synthesized using the PrimeScript III RT–PCR Kit (TaKaRa). Real‐time qPCR (Fig 6A) was performed with Thunderbird SYBR qPCR Mix (TOYOBO) using 1 μl of cDNA diluted five times as a template. Three experimental replicates for each genotype were analyzed. For RT–PCR (Appendix Fig S1B), 1 μl of 20‐fold diluted cDNA was used for PCR as a template. PCR products were digested with *Pml* I, which digests only *VANC21* transcribed from *Hi*TG. The primers used for RT–PCR are listed in Appendix Table S1.

### Small RNA analysis

Publicly available small RNA data from WT and *ddcc* (Stroud *et al*, 2014a; Data ref: Stroud *et al*, 2014b) were trimmed by Trimmomatic and mapped to TAIR10 reference genome using Bowtie (Langmead *et al*, 2009) with the following parameters: ‐n 0 ‐M 1 ‐‐best. 24‐nt mapped reads were extracted using SAMtools (Li *et al*, 2009) and visualized with Integrated Genome Viewer (IGV; Robinson *et al*, 2011).

## Author contributions

**Taku Sasaki:** Conceptualization; resources; data curation; formal analysis; funding acquisition; validation; investigation; visualization; methodology; writing – original draft; writing – review and editing. **Kae Kato:** Investigation. **Aoi Hosaka:** Investigation. **Yu Fu:** Resources. **Atsushi Toyoda:** Investigation. **Asao Fujiyama:** Investigation. **Yoshiaki Tarutani:** Investigation. **Tetsuji Kakutani:** Conceptualization; funding acquisition; writing – original draft; writing – review and editing.

## Disclosure and competing interests statement

The authors declare that they have no conflict of interest.

## Supporting information



Appendix

Source Data for Figure 1

Source Data for Figure 2

Source Data for Figure 3

Source Data for Figure 4

Source Data for Figure 5

Source Data for Figure 6

## Data Availability

The WGBS data obtained in this study are deposited in DDBJ under the accession number DRA014577.

## Footnotes

^#^
Correction added on 3 August 2023, after first online publication: “immobilized” has been corrected to “mobilized”.
