# Supplementary material for: Arms race between anti‐silencing and RdDM in noncoding regions of transposable elements
Source: EMBO Rep. 2023 Jun 5;24(8):e56678. doi: 10.15252/embr.202256678 (PMC10398659; doi:10.15252/embr.202256678)
Supplement: Supplementary file 1 — Appendix [file EMBR-24-e56678-s003.pdf]

Appendix – Table of contents

Appendix Figure S1 2
Appendix Figure S2 3
Appendix Figure S3 4
Appendix Figure S4 5
Appendix Figure S5 6
Appendix Figure S6 7
Appendix Figure S7 8
Appendix Figure S8 9
Appendix Figure S9 10
Appendix Figure S10 11
Appendix Figure S11 12
Appendix Figure S12 13
Appendix Table S1 14

Appendix Figure S1. Sasaki *et al*.

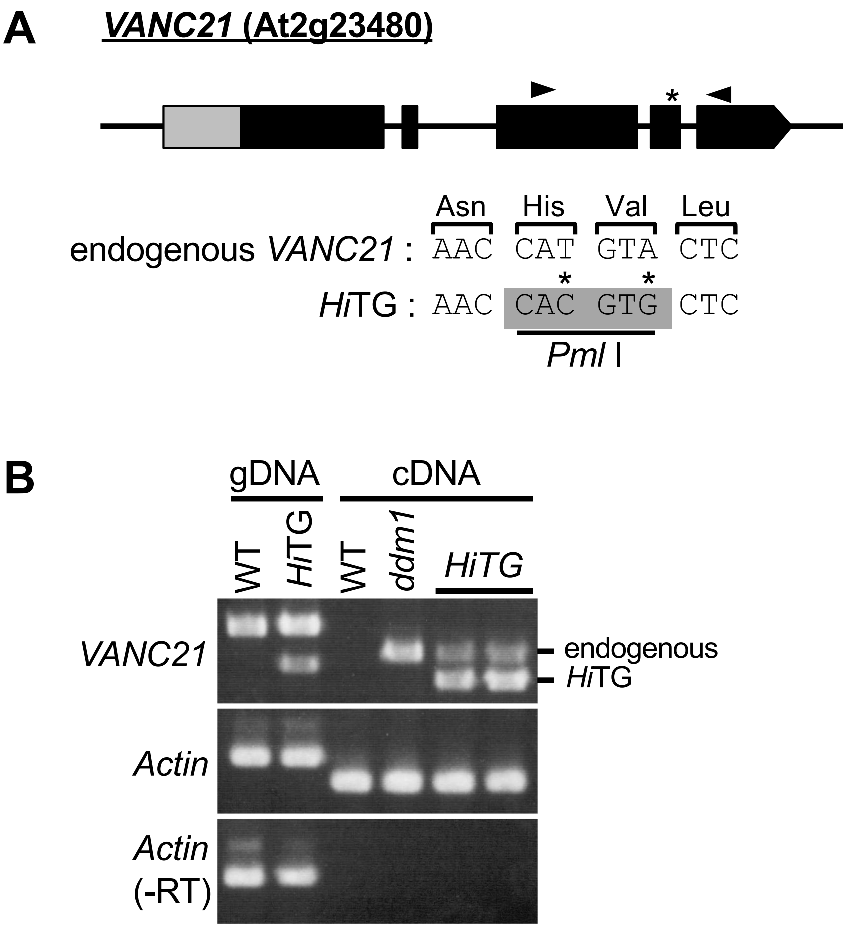

Appendix Figure S1. Activation of endogenous *Hi* by *Hi*TG.

(A) Schematic structure of *VANC21* and two synonymous mutations induced in *Hi*TG. These mutations created the recognition site of *Pml* I. Asterisks show the positions of mutations. Arrows indicate the positions of primers used for RT-PCR.
(B) Expression of endogenous and transgenic *VANC21* detected by RT-PCR followed by restriction digestion by *Pml* I.

Appendix Figure S2. Sasaki *et al*.

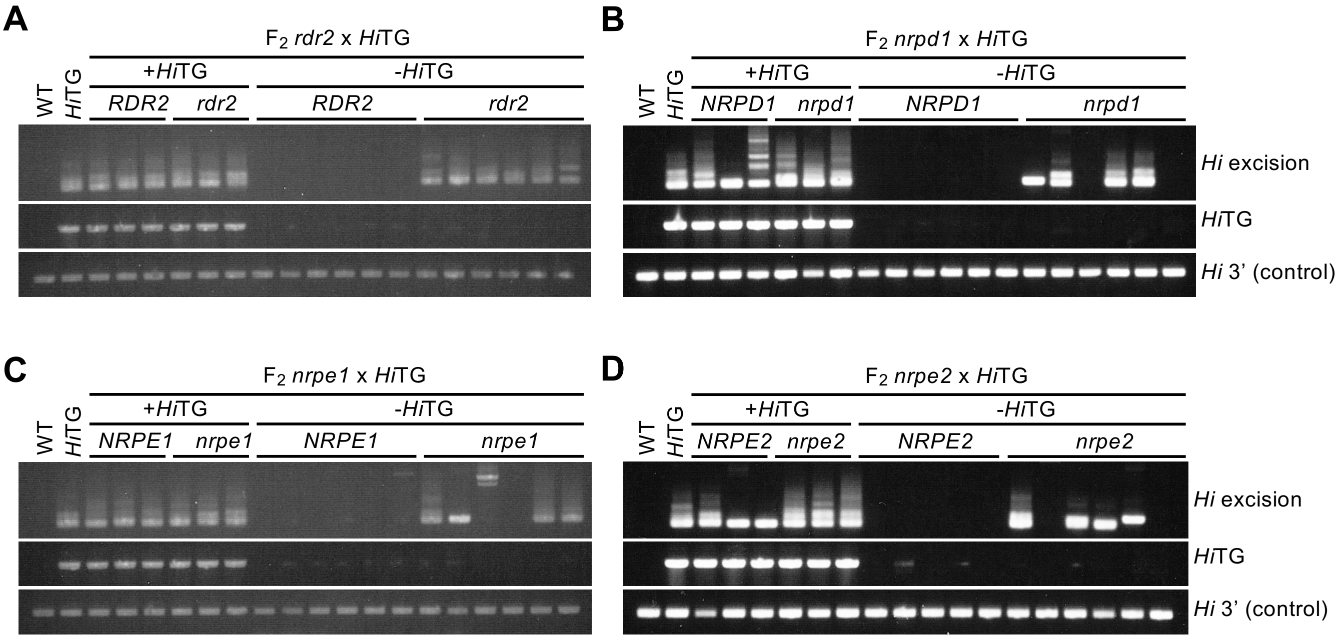

Appendix Figure S2. Mutation of RdDM components compromises the re-silencing of endogenous *Hi* when *Hi*TG has segregated away.

(A-D) Excision of an endogenous *Hi* in mutants of RdDM components, such as *rdr2* (A), *nrpd1* (B), *nrpe1* (C), and *nrpd/e2* (D).

Appendix Figure S3. Sasaki *et al*.

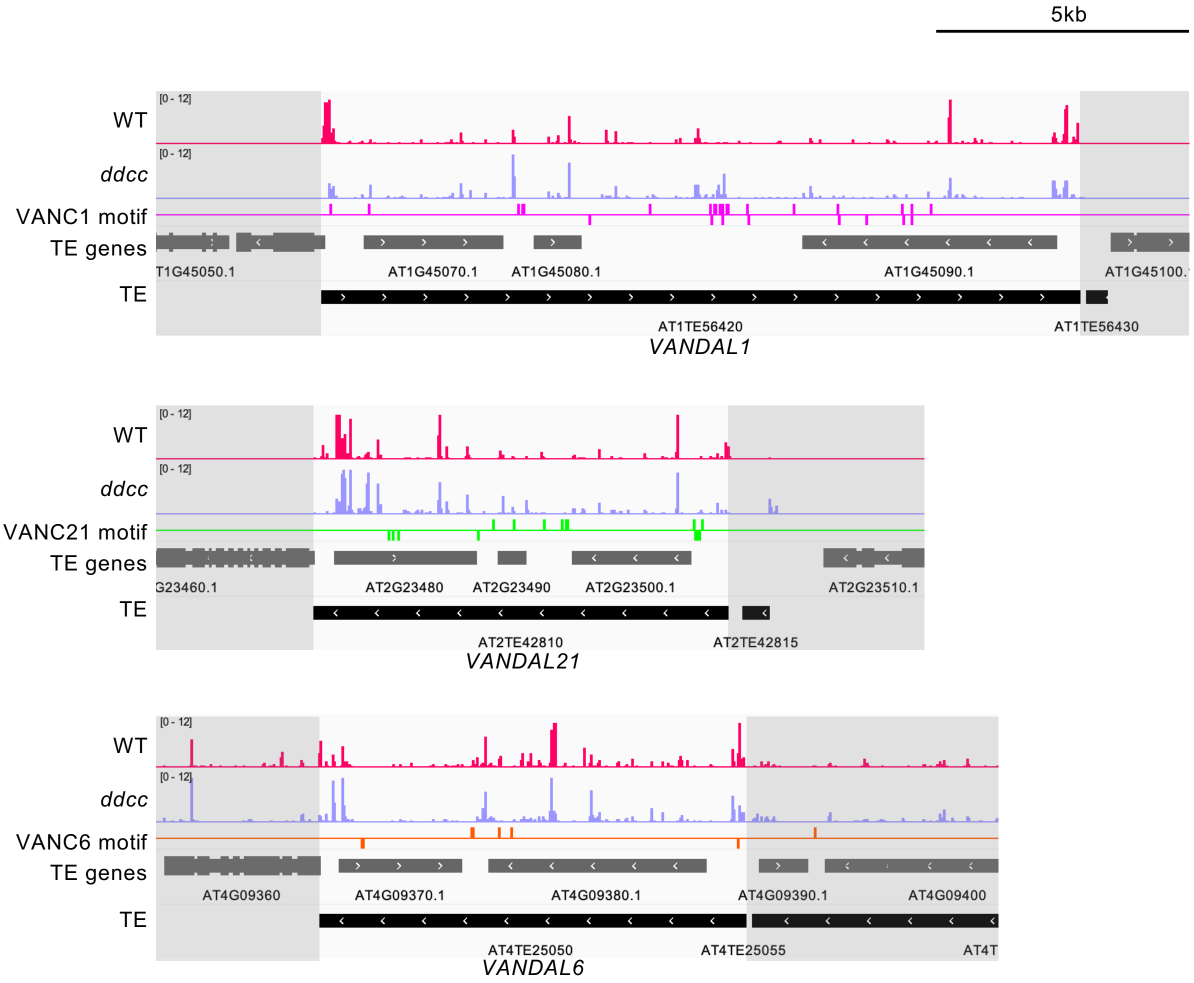

Appendix Figure S3. siRNA accumulation in *VANDAL* TEs.

24-nt small RNA accumulation in *VANDAL*s in WT and *ddcc* mutant is shown.

Appendix Figure S4. Sasaki *et al*.

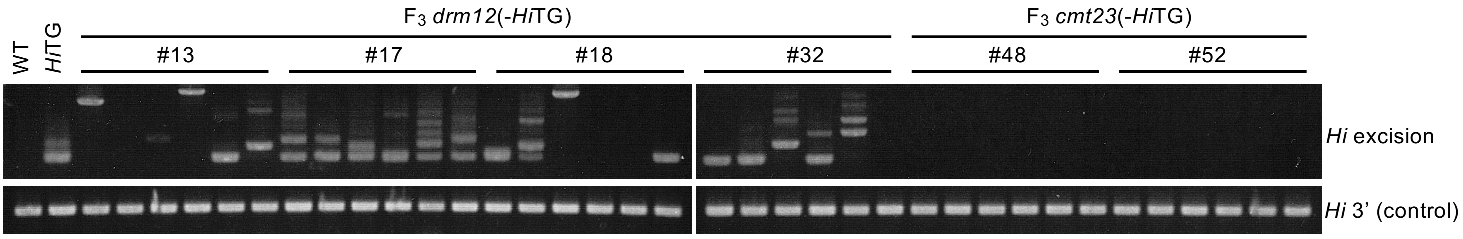

Appendix Figure S4. RdDM-independent re-silencing was promoted in the F3 generation.

Excision of an endogenous *Hi* in F3 plants of self-pollinated offspring of *drm12* (-*Hi*TG) and *cmt23* (-*Hi*TG) shown in Fig 2.

Appendix Figure S5. Sasaki *et al*.

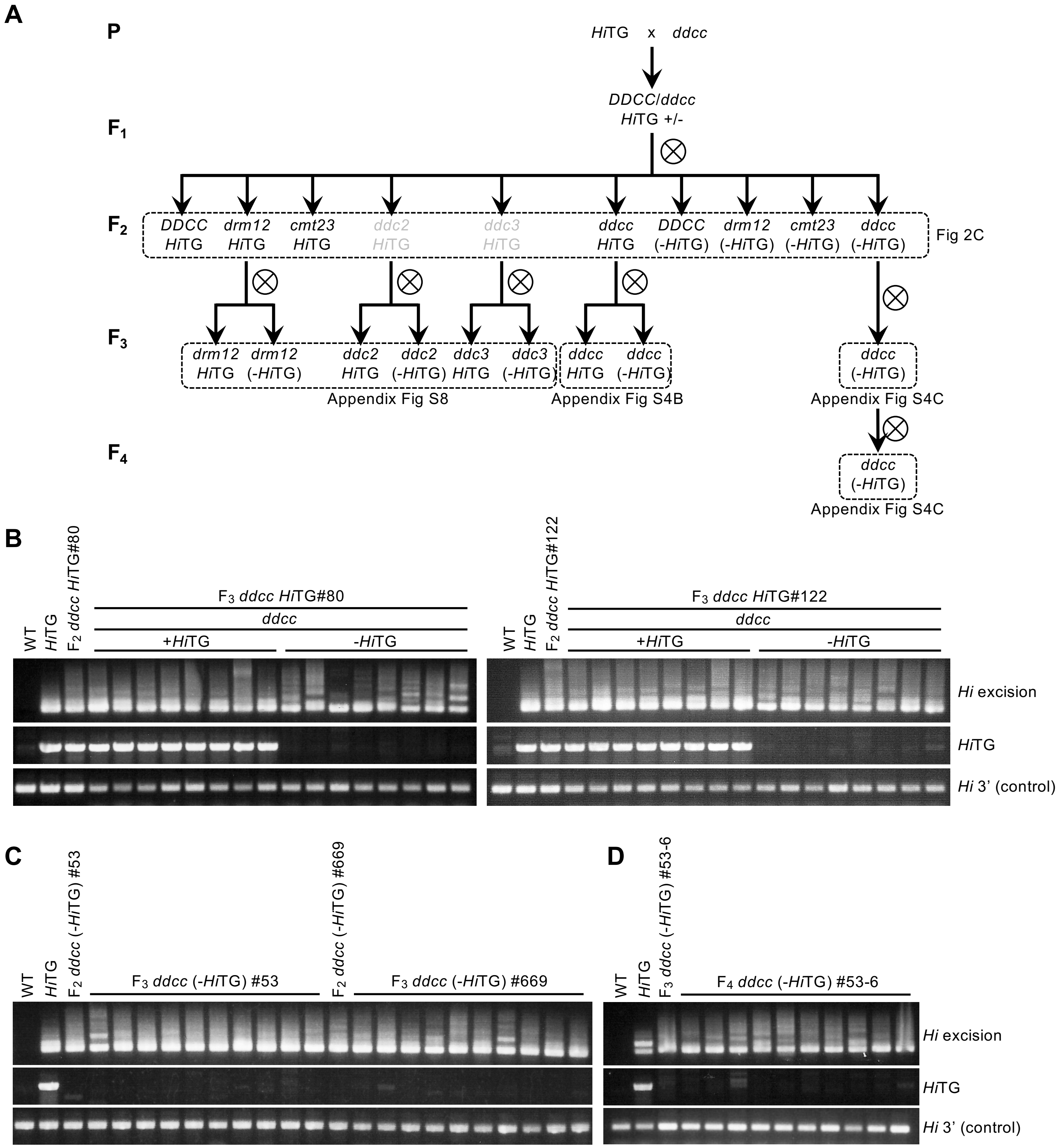

Appendix Figure S5. RdDM-independent re-silencing of *Hi* activity was repressed in the *ddcc* mutant background.

(A) Pedigree for materials used for analyses.
(B) Excision of endogenous *Hi* in the *ddcc* mutant background. *ddcc* was fixed in the F2 generation, and excision in self-pollinated F3 plants with and without *Hi*TG was analyzed.
(C) Excision of endogenous *Hi* in F3 *ddcc* (-*Hi*TG) whose genotype was already fixed in F2.

Appendix Figure S6. Sasaki *et al*.

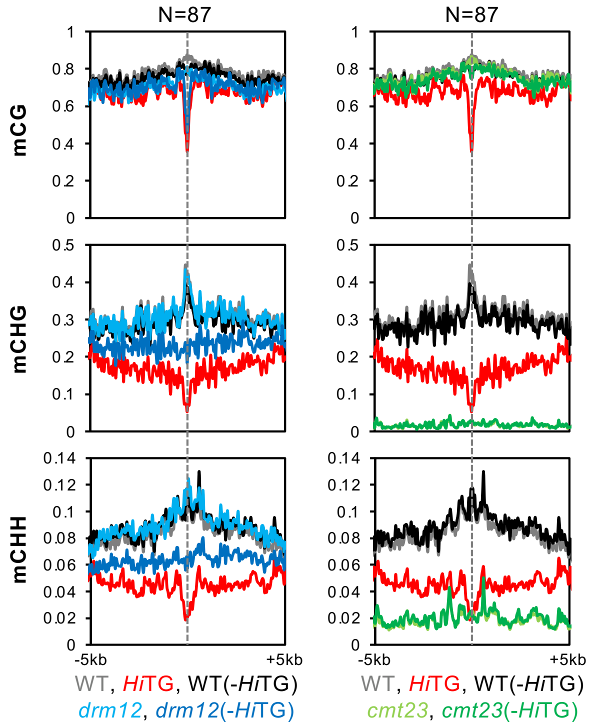

Appendix Figure S6. Target regions of RdDM and VANC21-binding regions detected by ChIP-seq overlap.

Patterns of DNA methylation in flanking regions of VANC21 targets when *Hi*TG was segregated away in *drm12* (left) and *cmt23* (right) mutant background. VANC21-binding regions detected by ChIP-seq (Hosaka et al. 2017) were used as VANC21 targets. Grey dashed lines indicate the points of VANC21 targets.

Appendix Figure S7. Sasaki *et al*.

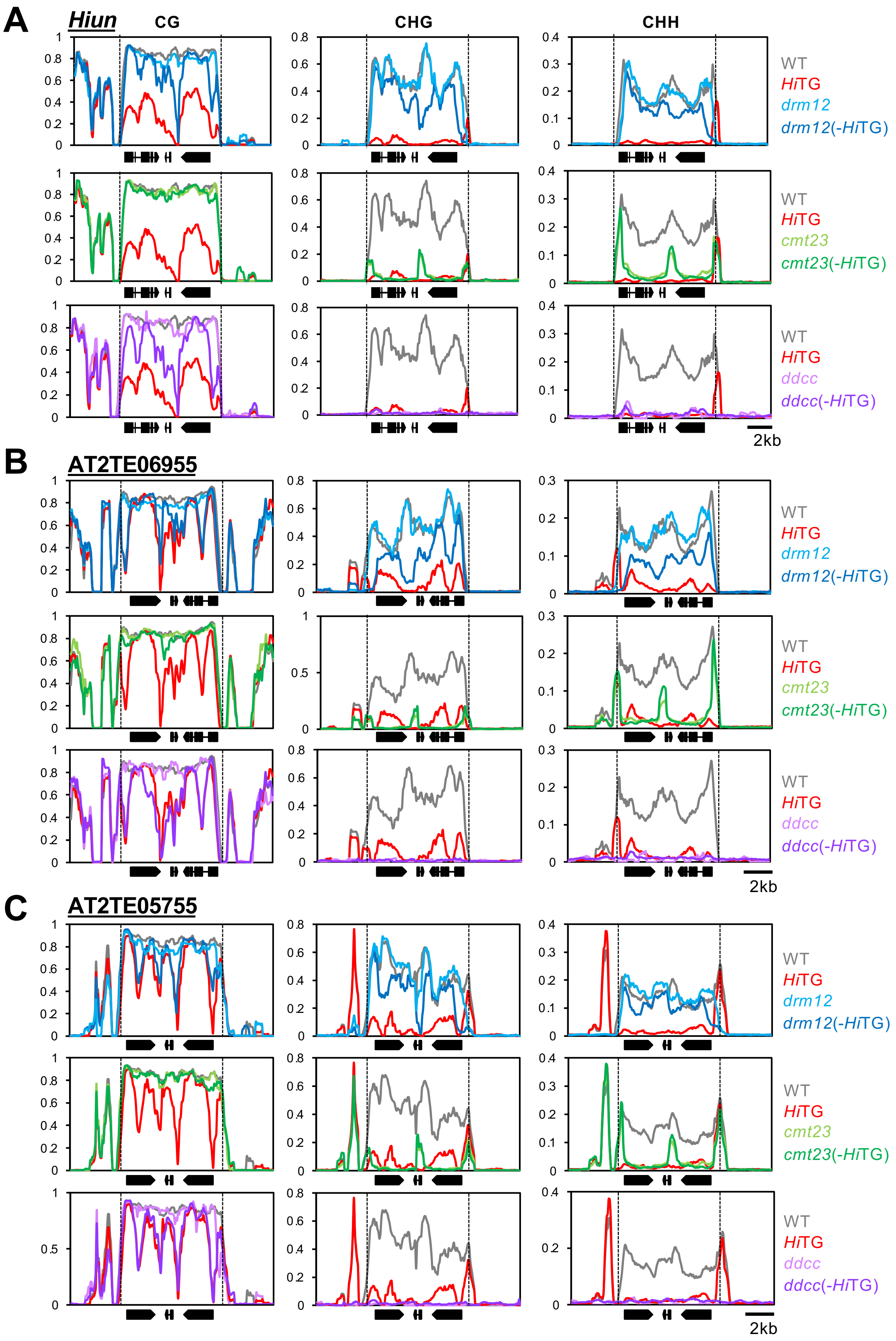

Appendix Figure S7. Patterns of DNA re-methylation in other *VANDAL21* TEs.

(A-C) Patterns of DNA methylation for *Hi* (A), *AT2TE06955* (B), and AT2TE05755 (C) in the *drm12*, *cmt23*, and *ddcc* mutant backgrounds.

Appendix Figure S8. Sasaki *et al*.

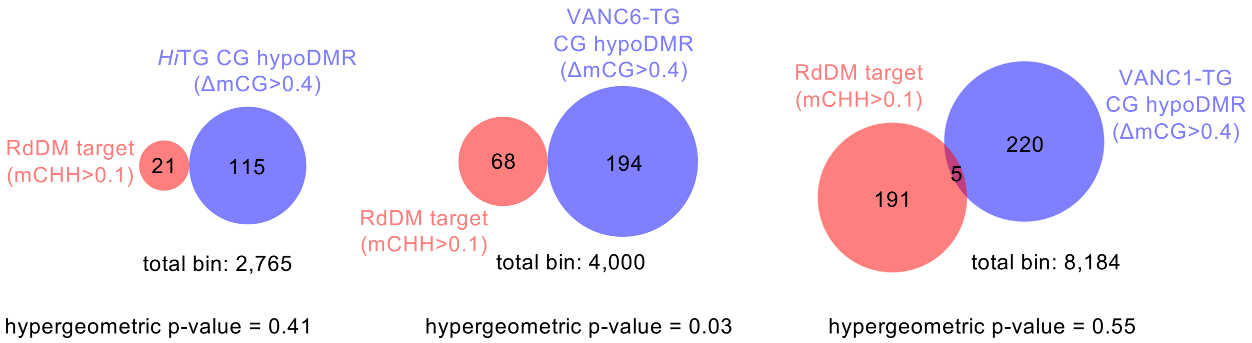

Appendix Figure S8. Control analysis of hypergeometric test using randomly selected regions.

Venn diagrams showing the overlap between targets of CG demethylation by VANCs and CHH methylation by RdDM using randomly selected regions. No significant enrichment of RdDM target regions was observed.

Appendix Figure S9. Sasaki *et al*.

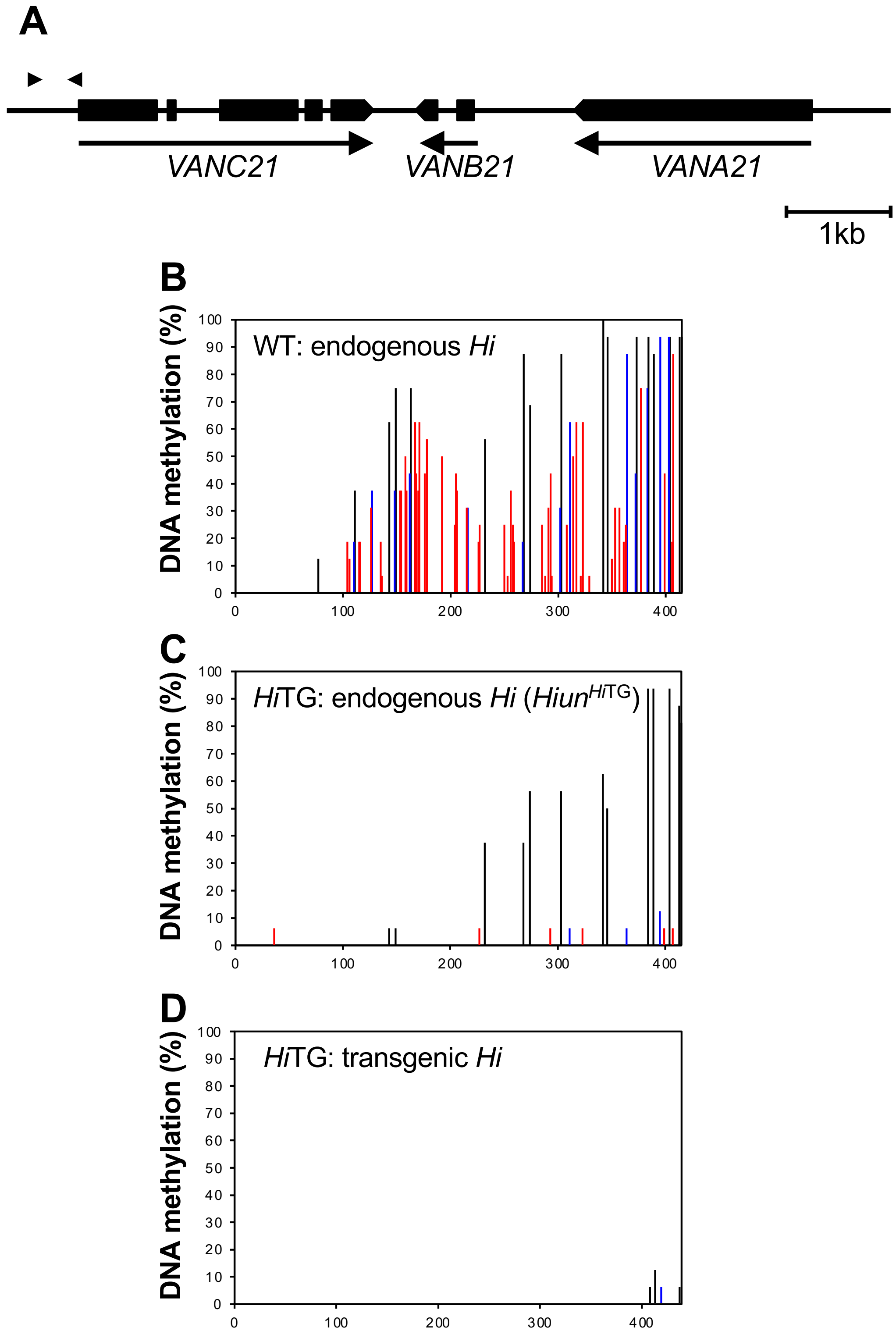

Appendix Figure S9. Differential DNA methylation among each activity status.

(A) Schematic structure of *Hiun*. Black boxes indicate exons. Positions of primers used for bisulfite sequencing are shown as black arrowheads.
(B-D) DNA methylation status of *Hi* in several genetic backgrounds detected by bisulfite sequencing.

Appendix Figure S10. Sasaki *et al*.

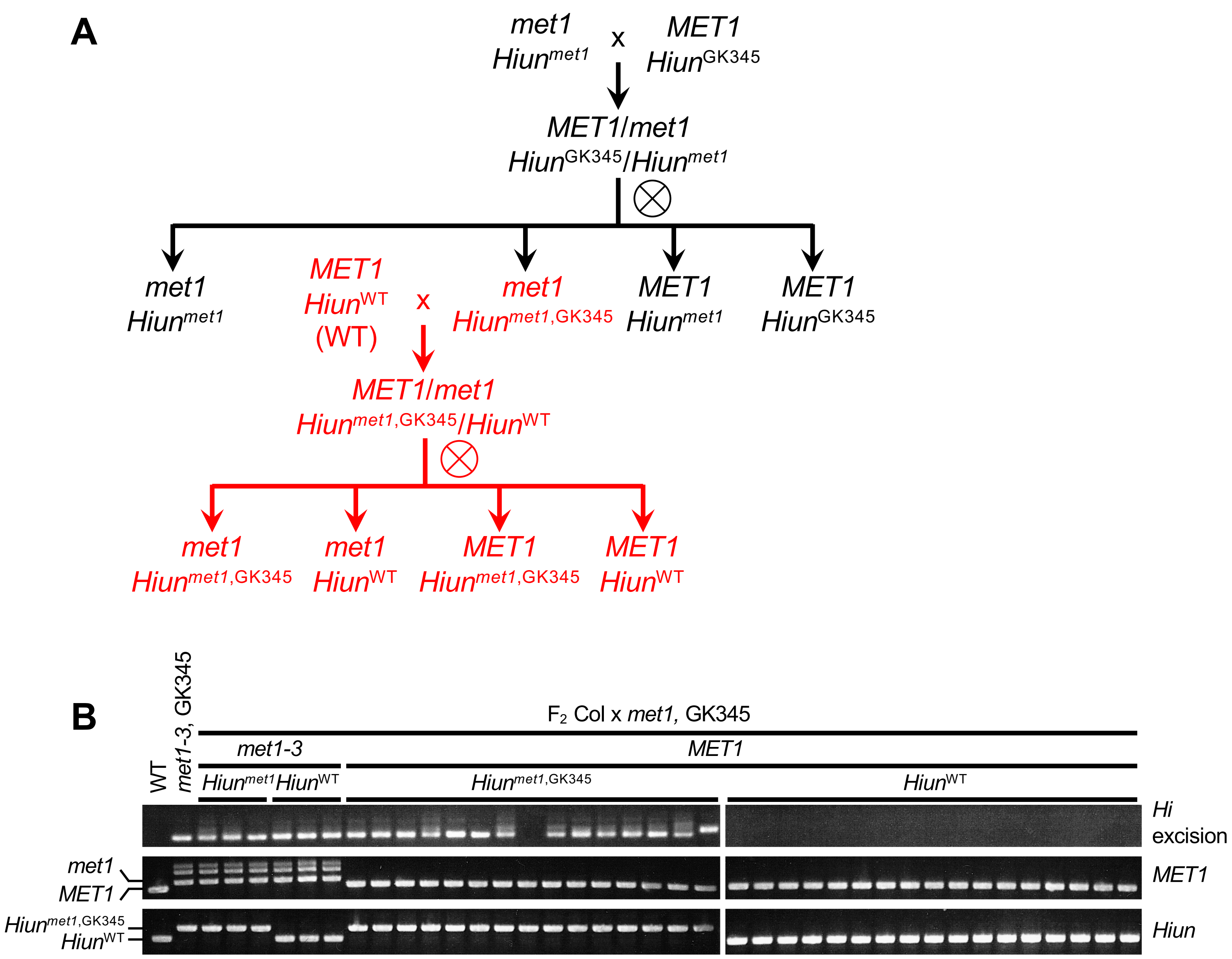

Appendix Figure S10. Importance of mCG for RdDM-mediated re-silencing of *Hi* (reciprocal cross of Fig. 5).

(A) Genetic scheme to generate CG hypomethylated *Hi*. Individuals shown in red were used in this experiment, and those in black were used in experiments shown in Figure 4.
(B) Excision of endogenous *Hi* in the F2 population of WT x *met1* GK345. Note that *Hiun*^{GK345} retained mobility despite its T-DNA insertion.

Appendix Figure S11. Sasaki *et al*.

A

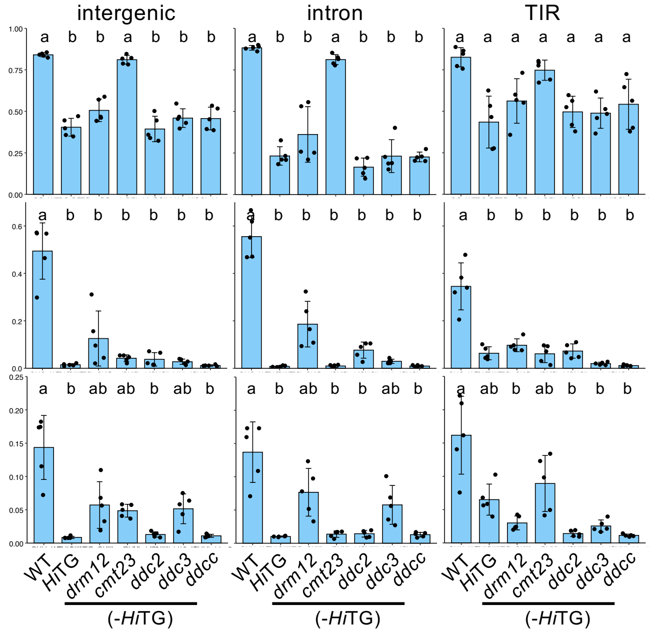

B

AT2TE05755			1264727-1271840
	TIR		1264727-1265117
	AT2G03990(VANC21-like)	exon	1265118-1265861, 1266277-1267104, 1267186-1267299
		intron	1265862-1266276, 1267105-1267185
	intergenic		1267300-1267761
	AT2G04000(VANB21-like)	exon	1267762-1267972, 1268158-1268337
		intron	1267973-1268157
	intergenic		1268338-1269098
	AT2G04010(VANA21-like)	exon	1269099-1271157
	TIR		1271158-1271840
AT2TE06955			1505385-1513550
	TIR		1505385-1506118
	AT2G04310(VANA21-like)	exon	1506119-1508509
	intergenic		1508510-1509376
	AT2G04320(VANB21-like)	exon	1509377-1509556, 1509734-1509910
		intron	1509557-1509733
	intergenic		1509911-1510449
	AT2G04330(VANC21-like)	exon	1510450-1510845, 1510933-1511094, 1511169-1511909, 1512423-1513142
		intron	1510846-1510932, 1511095-1511168, 1511910-1512422
	TIR		1513143-1513550
AT2TE20140			4861229-4869425
	TIR		4861229-4861655
	AT2G12060(VANC21-like)	exon	4861656-4862363, 4862878-4863612, 4863692-4863852, 4863929-4864288, 4864372-4864446
		intron	4862364-4862877, 4863613-4863691, 4863853-4863928, 4864289-4864371
	intergenic		4864447-4864906
	AT2G12066(VANB21-like)	exon	4864907-4865125, 4865303-4865482
		intron	4865126-4865302
	intergenic		4865483-4866379
	AT2G12083(VANA21-like)	exon	4866380-4868665
	TIR		4868666-4869425
AT3TE48070			11541283-11549498
	TIR		11541283-11542026
	AT3G29695(VANA21-like)	exon	11542027-11544487
	intergenic		11544488-11545356
	AT3G29700(VANB21-like)	exon	11545357-11545536, 11545714-11545932
		intron	11545537-11545713
	intergenic		11545933-11546269
	AT3G29710(VANC21-like)	exon	11546270-11546383, 11546467-11546826, 11546904-11547065, 11547140-11547880, 11548296-11549066
		intron	11546384-11546466, 11546827-11546903, 11547066-11547139, 11547881-11548295
	TIR		11549067-11549498
AT5TE49755			13960646-13968612
	TIR		13960646-13961410
	AT5G35791(VANA21-like)	exon	13961411-13963498
	intergenic		13963499-13964642
	AT5G35792(VANB21-like)	exon	13964643-13964822, 13965000-13965218
		intron	13964823-13964999
	intergenic		13965219-13965555
	AT5G35794(VANC21-like)	exon	13965556-13965669, 13965753-13966112, 13966190-13966351, 13966425-13967165, 13967483-13968112
		intron	13965670-13965752, 13966113-13966189, 13966352-13966424, 13967166-13967482
	TIR		13968113-13968612

Appendix Figure S11. Effect of each mCH DNA methyltransferase for re-methylation of *VANDAL21* TEs.

(A) DNA methylation status of intergenic regions, introns, and TIRs of *VANDAL21* TEs. The average values and standard deviations for each region are shown. One-way ANOVA with Tukey's multiple comparisons test was used to determine the significant differences ($p<0.01$).

(B) List of *VANDAL21* TEs and their annotation of coding genes used in (A) and Figure 6B.

Appendix Figure S12. Sasaki *et al*.

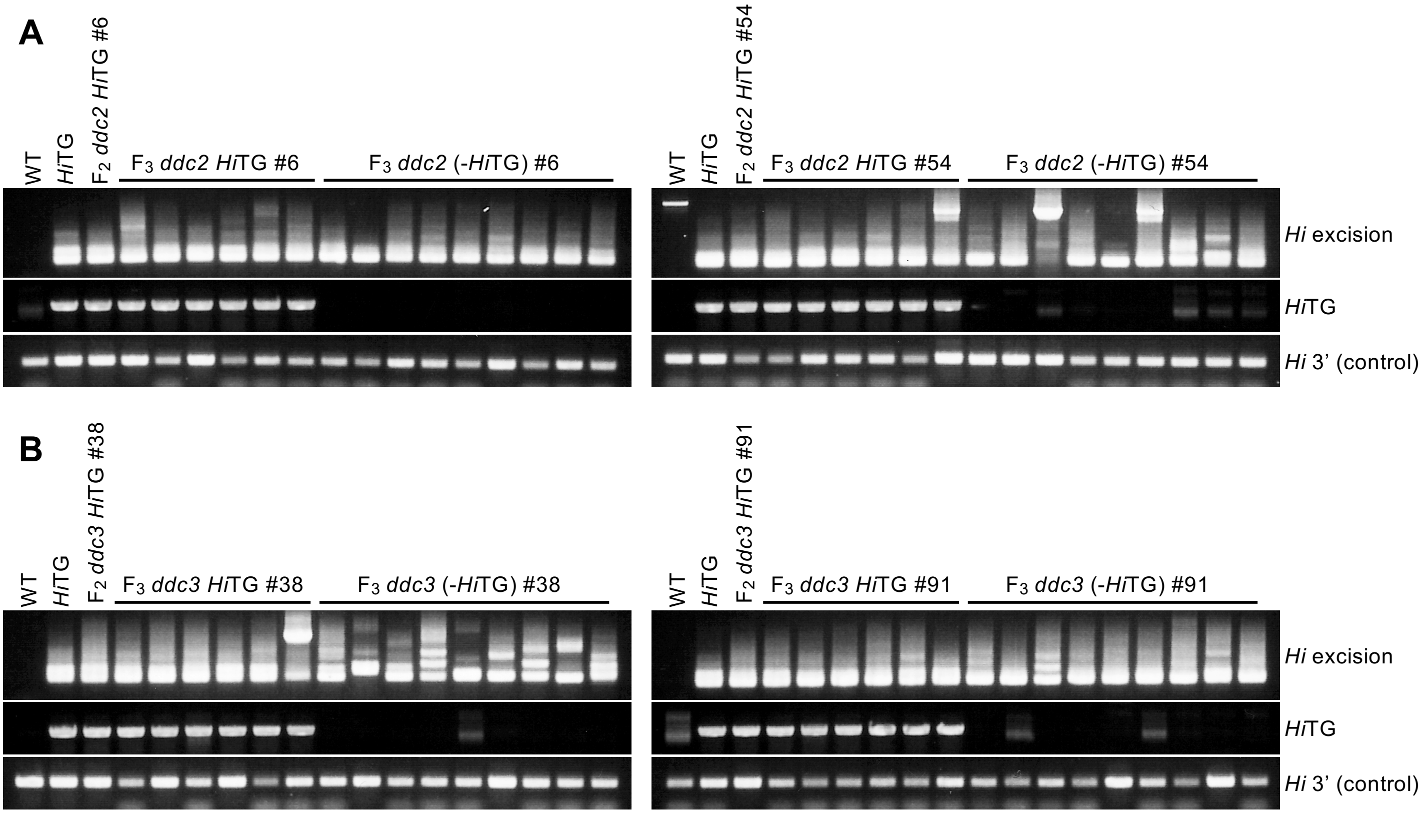

Appendix Figure S12. Effect of CMT2- and CMT3-dependent gene-body mCH for re-silencing of *Hi*.

(A, B) Excision of endogenous *Hi* in *ddc2* (A) and *ddc3* (B) mutant background. The pedigree for materials is shown in Appendix Figure S5A.

Appendix Table S1. Sasaki *et al*.

Appendix Table S1. Primers used in this study.

name	sequence (5'-3')	purpose
At2g23500F(-1)	ACG AGC AGA AAA CAT GCC ACC A	*Hi* excision (1st PCR)
At2g23500RV1	TGC TCT AAA CAT TGC CTG AAG C	(Fu et al. 2013)
At2g23500F2	CGA CGA GCT ACG TTA CTG GG	*Hi* excision (2nd PCR)
At2g23500RV3	AGT CTA TTC ACC ATC GCC TAG TT	(Fu et al. 2013)
pPZP_35SHPT_Nos	GCG GGC AGT TCG GTT TCA GGC	genotyping
pPZP_LB35S	GAC AAC TTA ATA ACA CAT TGC G	for *Hi*TG
345D06F	GCTA CGT TAC TGG GTA CTA CGA GG	
GK345_R2	AATA GGG AAT GTT GCA GTT GAA ACG G	genotyping for GK345
GK8474up	GGG CTA CAC TGA ATT GGT AGC TC	
At2g23500F(-1)	ACG AGC AGA AAA CAT GCC ACC A	3' region of *Hi*
Hi3'endF	GTC TTA ACA CAA ATC ATT AAG TG	(control)
HiC_BisF2	YTY GGT TYT GTG AGA ATA ATY GTY TGG	Bisulfite sequencing
HiC_BisR1	CTC CTT TCT TTT CCC TTR ACC TCC TCC AC	VANC21 side
pPZP_BisF1	GTA ATA YGA YTY AYT ATA GGG YGA ATT GGG	Bisulfite sequencing
HiC_BisR1	CTC CTT TCT TTT CCC TTR ACC TCC TCC AC	VANC21 side of *Hi*TG
VanAseqF1	CGT CAA GAA TTT CCT AAC AAG GC	RT-PCR
Van21seq10	TCG CCT TAG GAT ATC TCA ACT	for VANA21
RT23490f1	GAC CCC TAC TAC GAT GAT ATG	RT-PCR
RT23490RV1	CCA TAG GAT TAC GGA ATA CCA	for VANB21
Van21seq3	CTC AAG AGA ATA TGC AAA CTG A	RT-PCR
2g23480RT-PCRf2	CAC CTC GTT GCT AAC TTC ATC	for VANC21
VanCseqF3	AAG CAC ATC TAC CAC CTG CCT	RT-PCR for VANC21
VanCseqR3	GAC CAA GAC GCT TCA TCC AAC T	(Suppl Fig S1B)
ACT for	GCC ATC CAA GCT GTT CTC TC	RT-PCR
ACT rev	GGG CAT CTG AAT CTC TCA GC	for Actin
VANB21_qF1	TAG CAT TGT CGA GAC GCG AA	RT-qPCR for VANB21
VANB21_qR1	ATC CCA AAG TTT ACG GAT GTG C	(Fu et al. 2013)
VANC21_qF1	AGG ATG TGC AAG GTG AGT TTC A	RT-qPCR for VANC21
VANC21_qR1	ACTC CCG TGA TTT CAG CCA A	(Fu et al. 2013)
UBC_qF1	CTG CGA CTC AGG GAA TCT TCT AA	RT-qPCR for UBC
UBC_qR1	TTG TGC CAT TGA ATT GAA CCC	(Fu et al. 2013)
