# Supplementary figures and images for: Arms race between anti‐silencing and RdDM in noncoding regions of transposable elements
Source: EMBO Rep. 2023 Jun 5;24(8):e56678. doi: 10.15252/embr.202256678 (PMC10398659; doi:10.15252/embr.202256678)

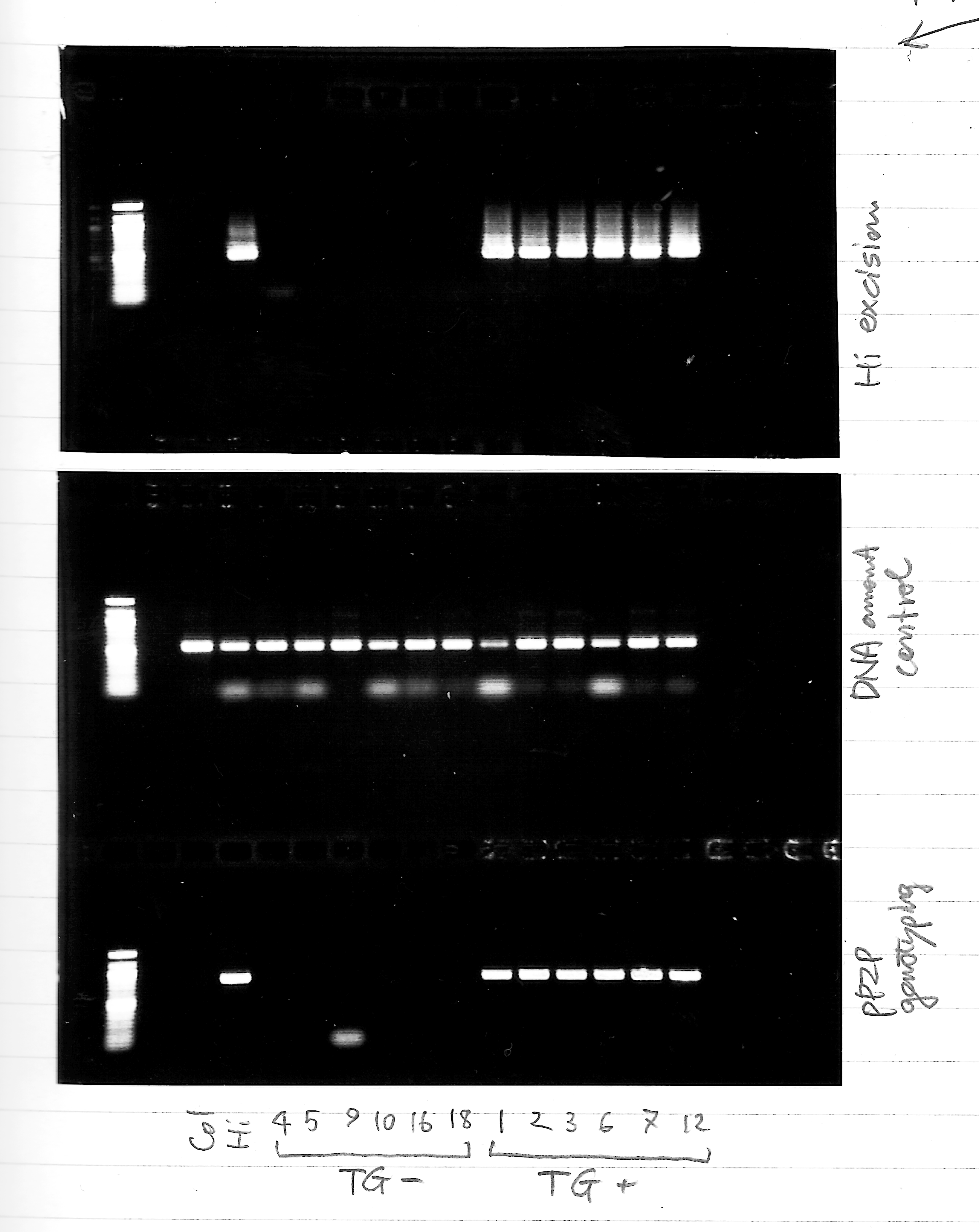

Supplement: Supplementary file 2 — Source Data for Figure 1 [file EMBR-24-e56678-s008.zip › SourceData_1A.tiff]

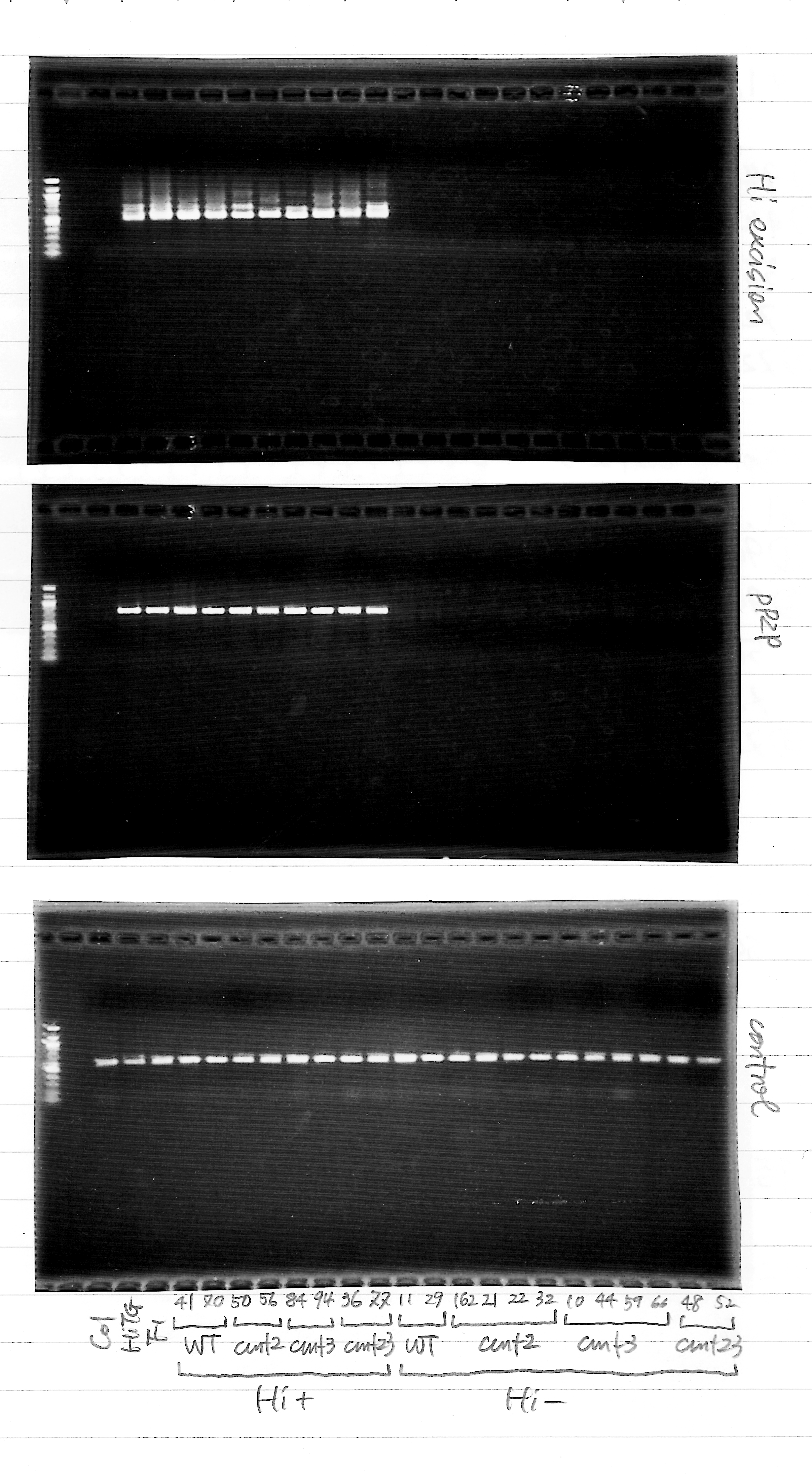

Supplement: Supplementary file 3 — Source Data for Figure 2 [file EMBR-24-e56678-s007.zip › SourceData_2A.tiff]

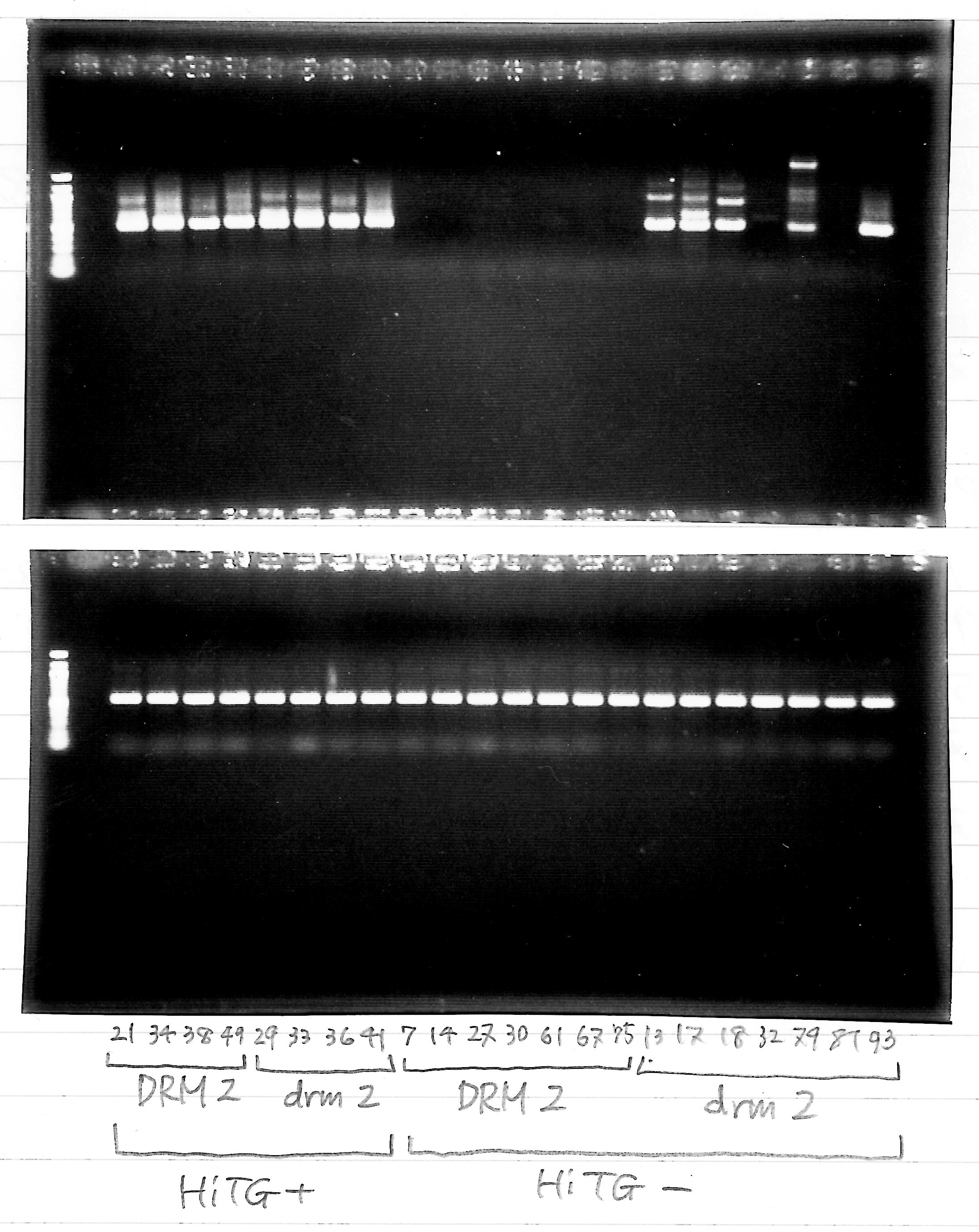

Supplement: Supplementary file 3 — Source Data for Figure 2 [file EMBR-24-e56678-s007.zip › SourceData_2B_1.tiff]

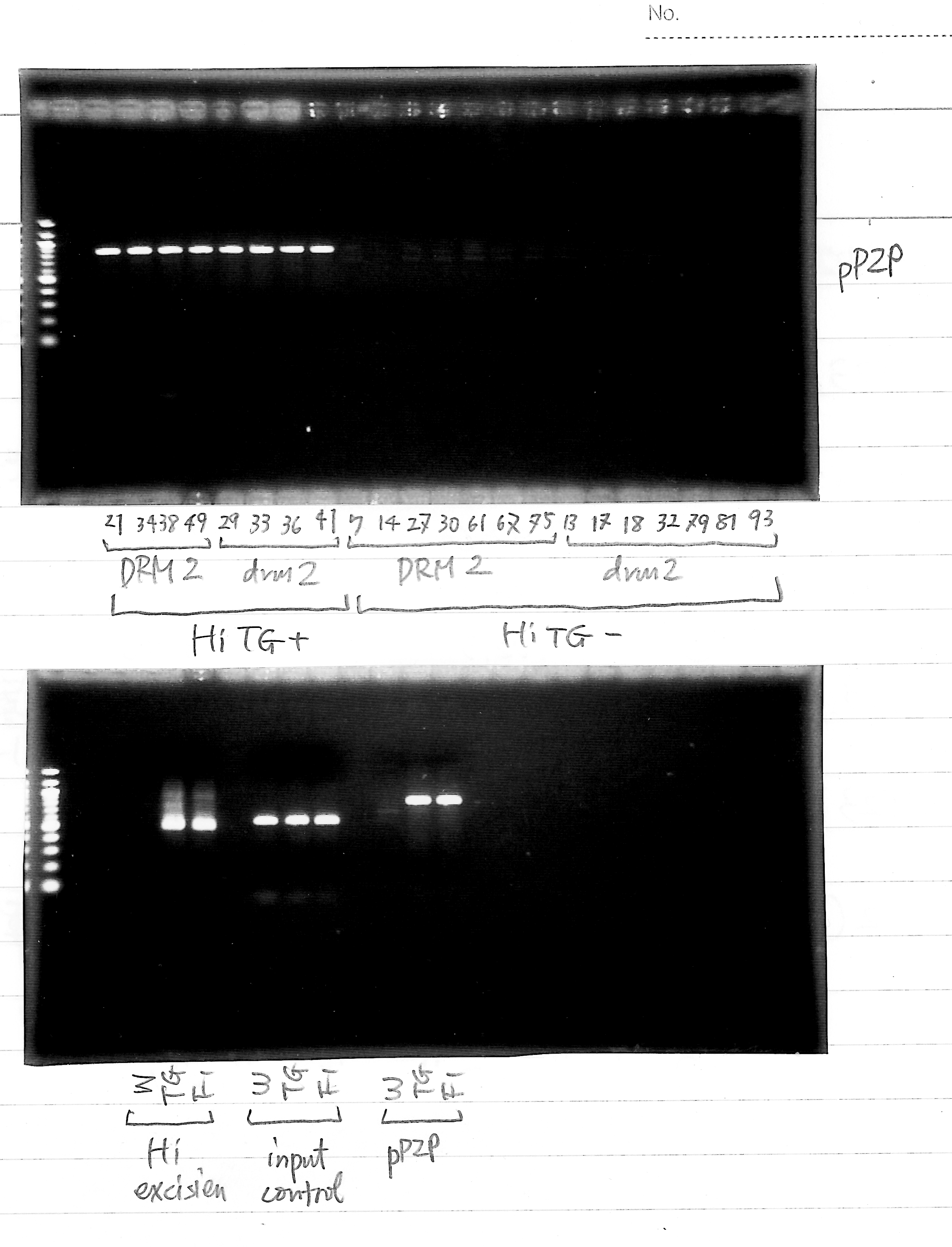

Supplement: Supplementary file 3 — Source Data for Figure 2 [file EMBR-24-e56678-s007.zip › SourceData_2B_2.tiff]

*Hi* excision

*HTG*

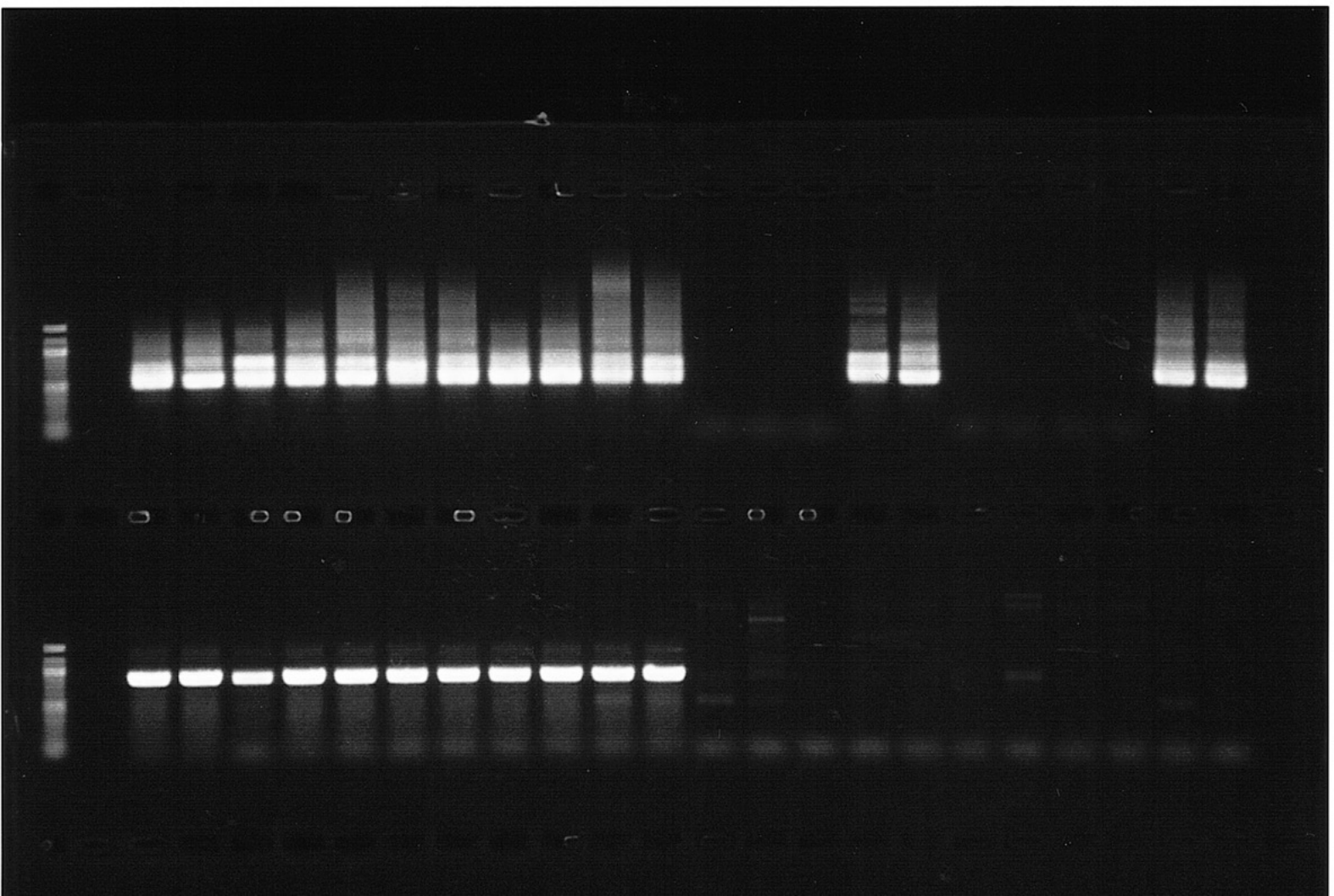

Supplement: Supplementary file 3 — Source Data for Figure 2 [file EMBR-24-e56678-s007.zip › SourceData_2C_1.pdf]

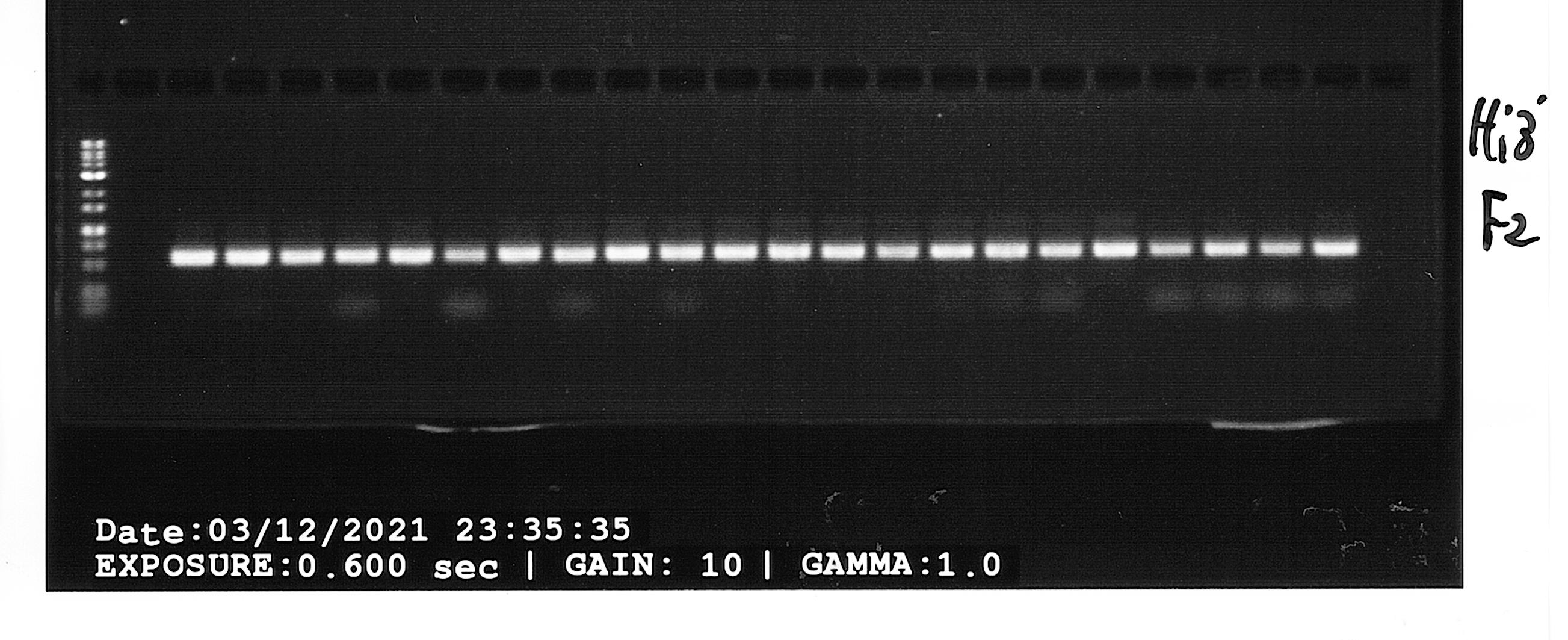

Supplement: Supplementary file 3 — Source Data for Figure 2 [file EMBR-24-e56678-s007.zip › SourceData_2C_2.tiff]

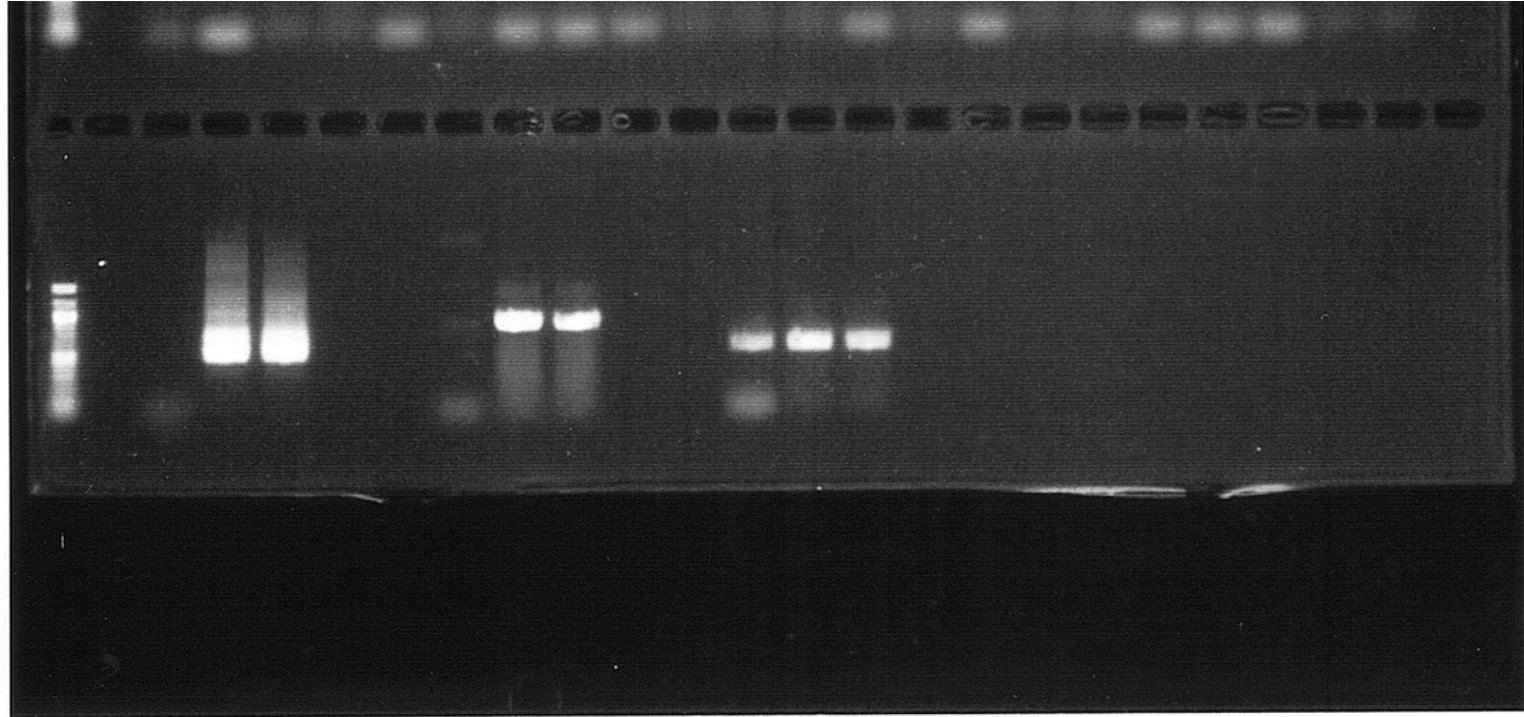

WT  
*HiTG*  
F1

*Hi excision*

WT  
*HiTG*  
F1

*HiTG*

WT  
*HiTG*  
F1

*Hi 3'*

Supplement: Supplementary file 3 — Source Data for Figure 2 [file EMBR-24-e56678-s007.zip › SourceData_2C_3.pdf]

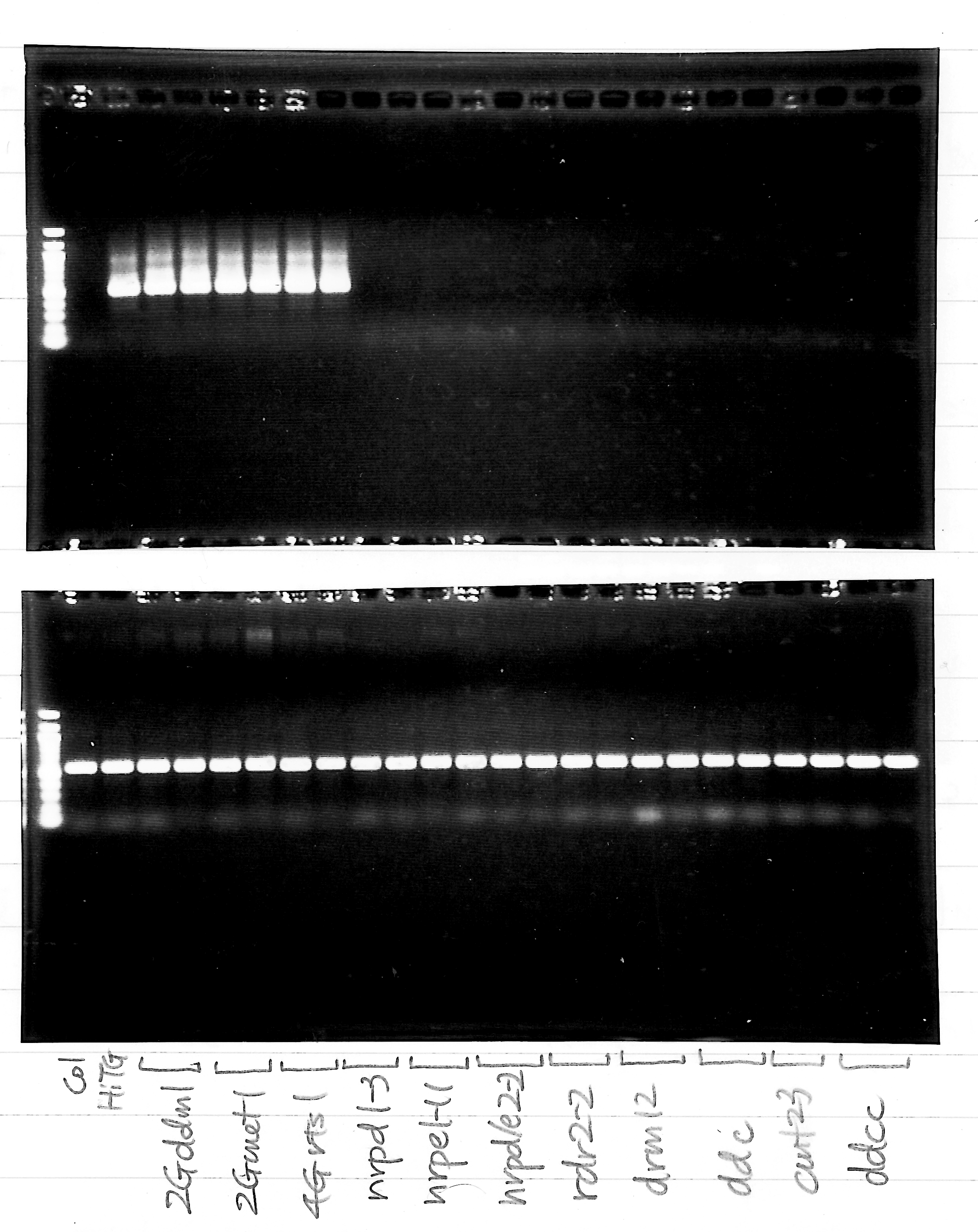

Supplement: Supplementary file 3 — Source Data for Figure 2 [file EMBR-24-e56678-s007.zip › SourceData_2D.tiff]

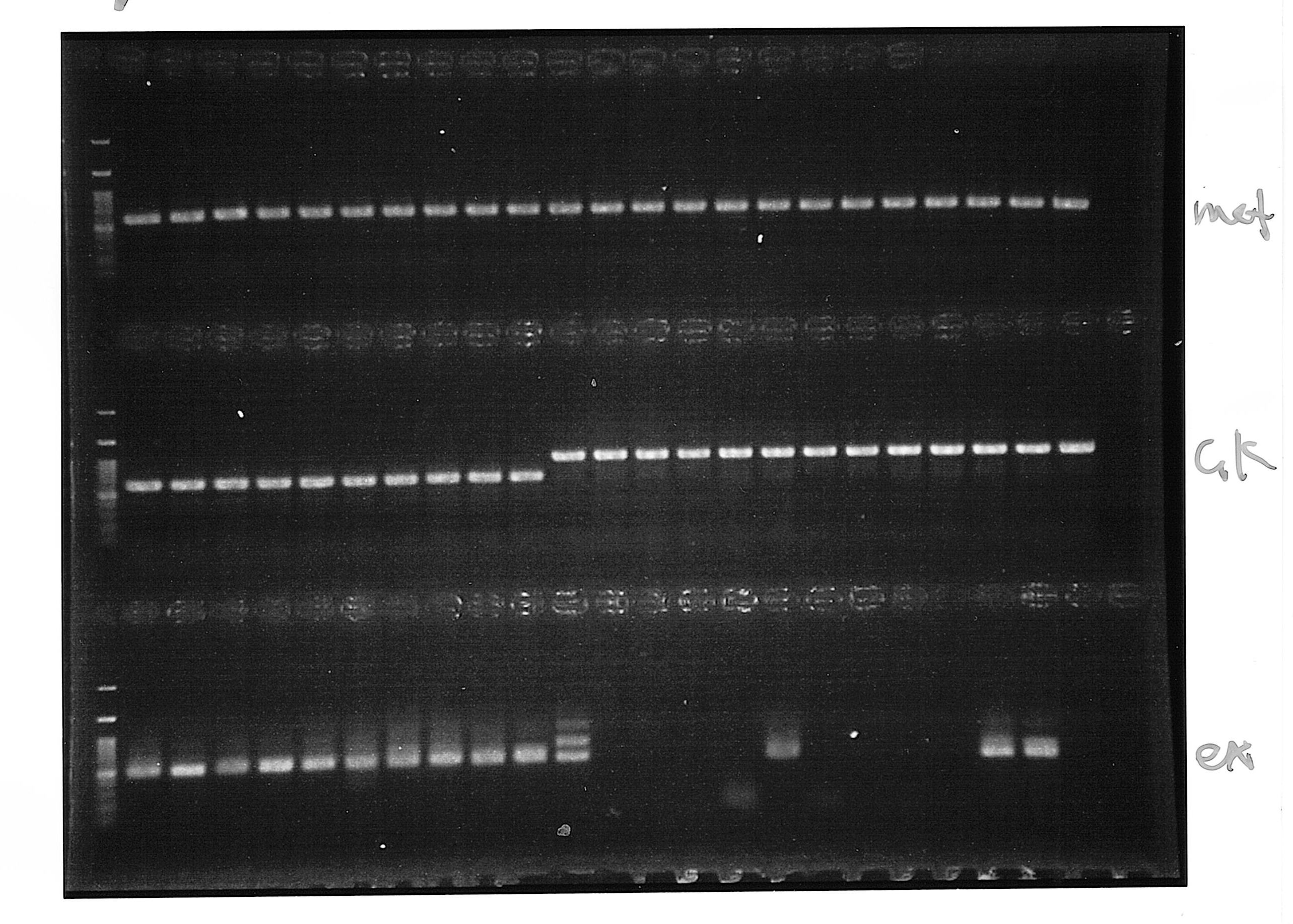

Supplement: Supplementary file 6 — Source Data for Figure 5 [file EMBR-24-e56678-s006.zip › SourceData_5C_2.tiff]

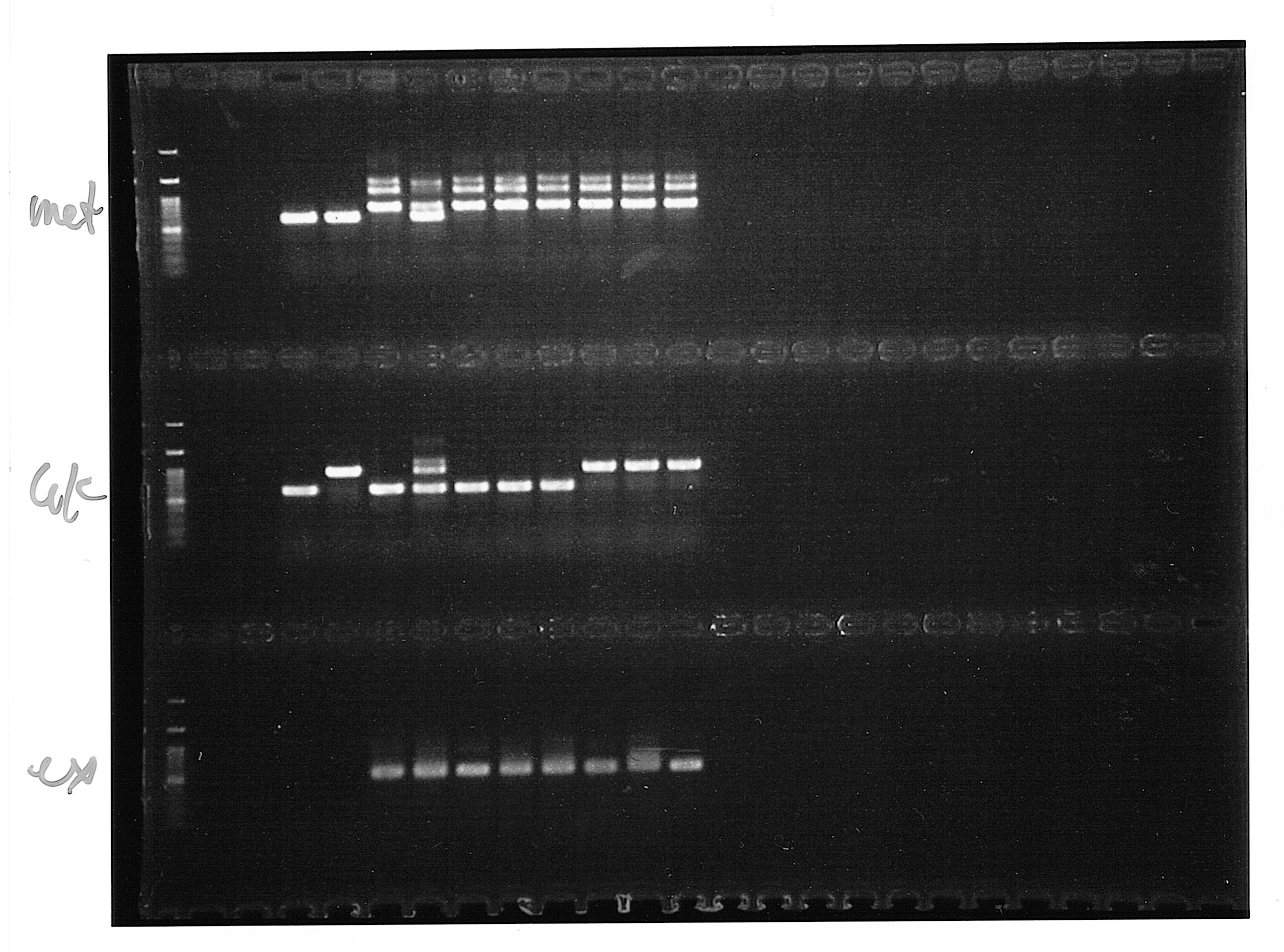

Supplement: Supplementary file 6 — Source Data for Figure 5 [file EMBR-24-e56678-s006.zip › SourceData_5C_1.tiff]
