# Supplementary material for: Arms race between anti‐silencing and RdDM in noncoding regions of transposable elements
Source: EMBO Rep. 2023 Jun 5;24(8):e56678. doi: 10.15252/embr.202256678 (PMC10398659; doi:10.15252/embr.202256678)
Supplement: Supplementary file 7 — Source Data for Figure 6 [file EMBR-24-e56678-s002.zip › SourceData_6C_1.pdf]

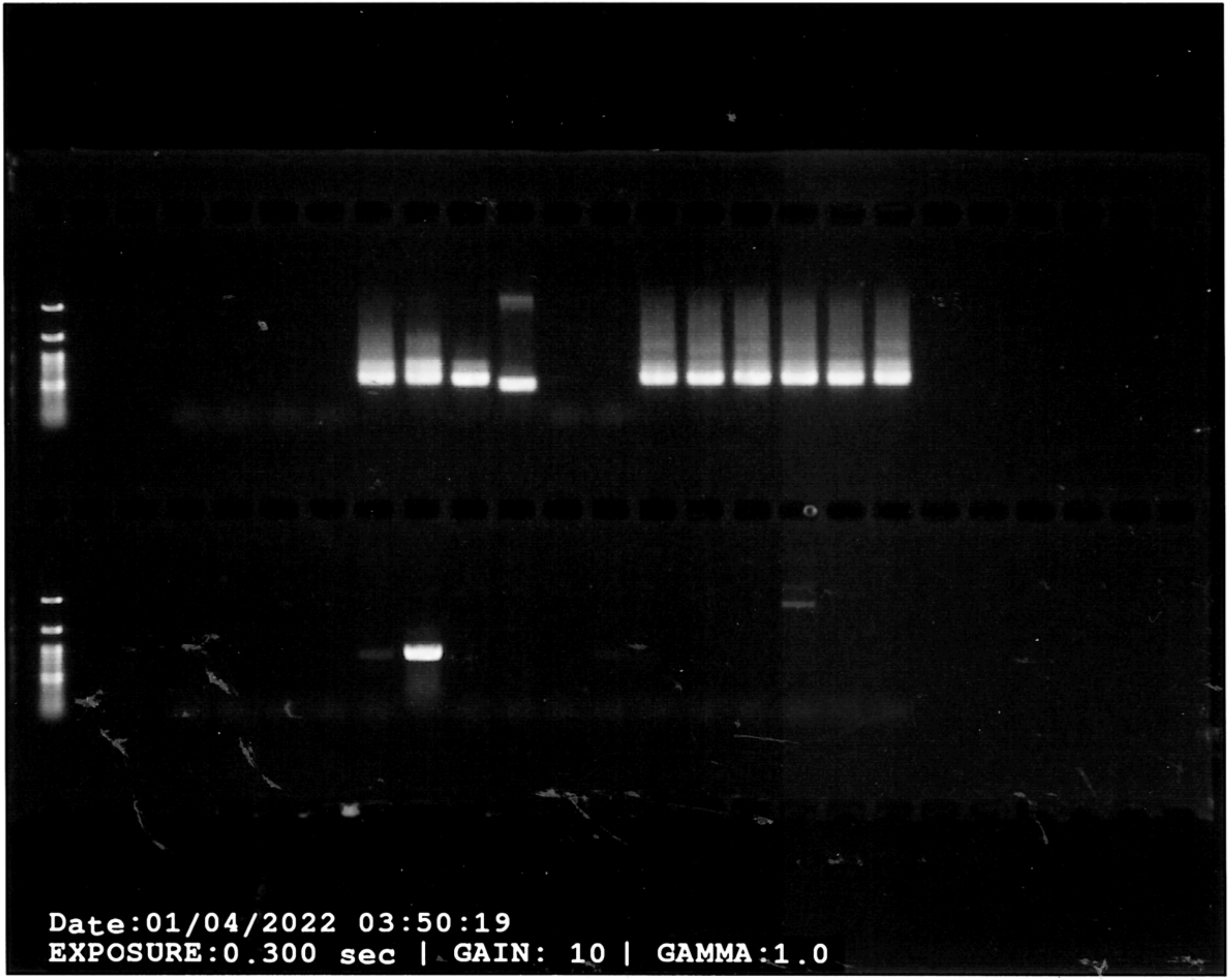

Hi excision

*Hi*TG
