# Supplementary material for: Arms race between anti‐silencing and RdDM in noncoding regions of transposable elements
Source: EMBO Rep. 2023 Jun 5;24(8):e56678. doi: 10.15252/embr.202256678 (PMC10398659; doi:10.15252/embr.202256678)
Supplement: Supplementary file 8 [file EMBR-24-e56678-s005.pdf]

EMBO reports

Arms race between anti-silencing and RdDM in noncoding regions of transposable elements

Taku Sasaki, Kae Kato, Aoi Hosaka, Yu Fu, Atsushi Toyoda, Asao Fujiyama, Yoshiaki Tarutani, and Tetsuji Kakutani
DOI: 10.15252/embr.202256678

Corresponding author(s): Taku Sasaki (taku.sasaki@bs.s.u-tokyo.ac.jp) , Tetsuji Kakutani (tkak@bs.s.u-tokyo.ac.jp)

Review Timeline:

Submission Date:	17th Dec 22
Editorial Decision:	25th Jan 23
Revision Received:	21st Apr 23
Editorial Decision:	7th May 23
Revision Received:	12th May 23
Accepted:	19th May 23

Editor: Esther Schnapp

Transaction Report:

(Note: With the exception of the correction of typographical or spelling errors that could be a source of ambiguity, letters and reports are not edited. Depending on transfer agreements, referee reports obtained elsewhere may or may not be included in this compilation. Referee reports are anonymous unless the Referee chooses to sign their reports.)

1st Editorial Decision **25th Jan 2023**

Dear Dr. Sasaki,

Thank you for the submission of your manuscript to EMBO reports. We have now received the full set of referee reports that is pasted below.

As you will see, the referees acknowledge that your findings are potentially interesting. However, they also have several suggestions for how the study should be improved and strengthened. Most suggestions regard textual changes, and the manuscript does need to be thoroughly re-written and the data need to be more carefully discussed. Referees 1 and 3 also ask for some more experiments including controls. I understand that point 7 by referee 1 is optional and that in the absence of new data the claims can be toned down. Point 9 by referee 1 should ideally be addressed experimentally. Please let me know how you plan to proceed with the revisions, so that I can discuss any questions you might have with the referees beforehand.

I would thus like to invite you to revise your manuscript with the understanding that the referee concerns must be fully addressed and their suggestions taken on board. Please address all referee concerns in a complete point-by-point response. Acceptance of the manuscript will depend on a positive outcome of a second round of review. It is EMBO reports policy to allow a single round of major revision only and acceptance or rejection of the manuscript will therefore depend on the completeness of your responses included in the next, final version of the manuscript.

We realize that it is difficult to revise to a specific deadline. In the interest of protecting the conceptual advance provided by the work, we recommend a revision within 3 months (27th Apr 2023). Please discuss the revision progress ahead of this time with the editor if you require more time to complete the revisions.

IMPORTANT NOTE: we perform an initial quality control of all revised manuscripts before re-review. Your manuscript will FAIL this control and the handling will be DELAYED if the following APPLIES:
1) A data availability section providing access to data deposited in public databases is missing. If you have not deposited any data, please add a sentence to the data availability section that explains that.
2) Your manuscript contains statistics and error bars based on n=2. Please use scatter blots in these cases. No statistics should be calculated if n=2.

When submitting your revised manuscript, please carefully review the instructions that follow below. Failure to include requested items will delay the evaluation of your revision.

1) a .docx formatted version of the manuscript text (including legends for main figures, EV figures and tables). Please make sure that the changes are highlighted to be clearly visible.

2) individual production quality figure files as .eps, .tif, .jpg (one file per figure). See https://wol-prod-cdn.literatumonline.com/pb-assets/embo-site/EMBOPress_Figure_Guidelines_061115-1561436025777.pdf for more info on how to prepare your figures.

3) We replaced Supplementary Information with Expanded View (EV) Figures and Tables that are collapsible/expandable online. A maximum of 5 EV Figures can be typeset. EV Figures should be cited as 'Figure EV1, Figure EV2" etc... in the text and their respective legends should be included in the main text after the legends of regular figures.

- For the figures that you do NOT wish to display as Expanded View figures, they should be bundled together with their legends in a single PDF file called *Appendix*, which should start with a short Table of Content. Appendix figures should be referred to in the main text as: "Appendix Figure S1, Appendix Figure S2" etc. See detailed instructions regarding expanded view here: <https://www.embopress.org/page/journal/14693178/authorguide#expandedview>

- Additional Tables/Datasets should be labeled and referred to as Table EV1, Dataset EV1, etc. Legends have to be provided in a separate tab in case of .xls files. Alternatively, the legend can be supplied as a separate text file (README) and zipped together with the Table/Dataset file.

4) a .docx formatted letter INCLUDING the reviewers' reports and your detailed point-by-point responses to their comments. As part of the EMBO Press transparent editorial process, the point-by-point response is part of the Review Process File (RPF), which will be published alongside your paper.

5) a complete author checklist, which you can download from our author guidelines <https://www.embopress.org/page/journal/14693178/authorguide>. Please insert information in the checklist that is also reflected in the manuscript. The completed author checklist will also be part of the RPF.

6) Please note that all corresponding authors are required to supply an ORCID ID for their name upon submission of a revised manuscript (<https://orcid.org/>). Please find instructions on how to link your ORCID ID to your account in our manuscript tracking system in our Author guidelines

<https://www.embopress.org/page/journal/14693178/authorguide#authorshipguidelines>

7) Before submitting your revision, primary datasets produced in this study need to be deposited in an appropriate public database (see https://www.embopress.org/page/journal/14693178/authorguide#datadeposition). Please remember to provide a reviewer password if the datasets are not yet public. The accession numbers and database should be listed in a formal "Data Availability" section placed after Materials & Method (see also https://www.embopress.org/page/journal/14693178/authorguide#datadeposition). Please note that the Data Availability Section is restricted to new primary data that are part of this study. * Note - All links should resolve to a page where the data can be accessed. *
If your study has not produced novel datasets, please mention this fact in the Data Availability Section.

8) At EMBO Press we ask authors to provide source data for the main manuscript figures. Our source data coordinator will contact you to discuss which figure panels we would need source data for and will also provide you with helpful tips on how to upload and organize the files.

9) Our journal also encourages inclusion of *data citations in the reference list* to directly cite datasets that were re-used and obtained from public databases. Data citations in the article text are distinct from normal bibliographical citations and should directly link to the database records from which the data can be accessed. In the main text, data citations are formatted as follows: "Data ref: Smith et al, 2001" or "Data ref: NCBI Sequence Read Archive PRJNA342805, 2017". In the Reference list, data citations must be labeled with "[DATASET]". A data reference must provide the database name, accession number/identifiers and a resolvable link to the landing page from which the data can be accessed at the end of the reference. Further instructions are available at https://www.embopress.org/page/journal/14693178/authorguide#referencesformat

10) Regarding data quantification (see Figure Legends: https://www.embopress.org/page/journal/14693178/authorguide#figureformat)

The following points must be specified in each figure legend:

- the name of the statistical test used to generate error bars and P values,

- the number (n) of independent experiments (please specify technical or biological replicates) underlying each data point,

- the nature of the bars and error bars (s.d., s.e.m.),

- If the data are obtained from n Program fragment delivered error ``Can't locate object method "less" via package "than" (perhaps you forgot to load "than"?) at //ejpvfs23/sites23b/embor_www/letters/embor_decision_revise_and_review.txt line 56.' 2, use scatter blots showing the individual data points.

Discussion of statistical methodology can be reported in the materials and methods section, but figure legends should contain a basic description of n, P and the test applied.

- Please also include scale bars in all microscopy images.

11) The journal requires a statement specifying whether or not authors have competing interests (defined as all potential or actual interests that could be perceived to influence the presentation or interpretation of an article). In case of competing interests, this must be specified in your disclosure statement. Further information: https://www.embopress.org/competing-interests

We would also welcome the submission of cover suggestions, or motifs to be used by our Graphics Illustrator in designing a cover.

As part of the EMBO publication's Transparent Editorial Process, EMBO reports publishes online a Review Process File (RPF) to accompany accepted manuscripts. This File will be published in conjunction with your paper and will include the referee reports, your point-by-point response and all pertinent correspondence relating to the manuscript.

You are able to opt out of this by letting the editorial office know (emboreports@embo.org). If you do opt out, the Review Process File link will point to the following statement: "No Review Process File is available with this article, as the authors have chosen not to make the review process public in this case."

I look forward to seeing a revised form of your manuscript when it is ready. Please use this link to submit your revision: https://embor.msubmit.net/cgi-bin/main.plex

Yours sincerely,

Esther Schnapp, PhD
Senior Editor
EMBO reports

Referee #1:

This is a review of the manuscript entitled "The arms race in noncoding regions of transposable elements: the evolution of ant-silencing and RNAi" submitted to EMBO Reports by Sasaki et al.
This work uses both a transgene system and mutant backgrounds to study the activation and resilencing of endogenous VANDAL transposable elements in Arabidopsis. The strength of the work is the ability to mechanistically separate the roles of CG DNA methylation and RNA-directed DNA methylation (RdDM), as well as the analysis of the co-regulation of particular non-coding regions of VANDAL elements by both RdDM and VANDAL-encoded anti-silencing proteins.

Major issues
1. In both the title and abstract, the term "evolution" is used. For example, the abstract says "Target motifs of VANC, in turn, evolved to escape RNAi". However, there is no sequence analysis of evolution in this work, and therefore the authors should not make evolutionary arguments. They can certainly say that this region is co-regulated, but saying "evolved to.." would take different sequence analysis experiments that were not performed here.
2. In the abstract and many times in the body, the authors refer to RNAi when they mean RdDM. This work did not test RDR6, SGS3 nor other RNAi proteins. It only tested RdDM proteins.
3. The Results remind me of the SPM and Mutator systems with autonomous and non-autonomous elements. The non-autonomous elements are full methylated, but then in the presence of the transposase protein from the active autonomous element, they become unmethylated, only to snap back to methylated when the autonomous element is segregated away. See the work of Damon Lisch using the Mutator system in maize. In this case, the methylated state can be thought of as the default, and only temporarily unmethylated in the presence of TE-binding protein. A similar phenomenon is likely happening here.
4. In the Results that describe Figure 2D, the authors describe that the TE is not mobilized in the ddcc mutant. Yet the data shows that the TE is not mobilized in any RdDM mutant. Please broaden the analysis and speculate what this means about the TE activation seen in the literature and field that occurs in any RdDM mutant. This is important because many researchers say that RdDM suppresses transposition, when actually there is very little evidence that it is important for transposition at all.
5. In the Results section "Targets of RdDM and VANC overlap", this overlap analysis of two datasets needs negative controls such as random regions of the genome or VANC regions. Pie charts is not the best way to show the overlap in this data. An analysis of observed overlap in these datasets compared to what would be expected by chance with datasets this size is more appropriate.
6. The last Results section is poorly written. It is unclear what this all means. Please write this section with effort to guide the reader of what these results mean.
7. In this same last Results section, the authors claim that the body of the VANC element is regulated differently. To make this claim, perform an analysis that compares the body, ends, coding, non-coding, etc... regions.
8. In Figure 3, it would help to add the methylation pattern of ddcc as a reference to compare drm12 to. Is all VANC methylation absent in ddcc?
9. Throughout the entire manuscript, it is clear that CG methylation controls VANC element expression. However, in Figure S6, the endogenous Hi element retains some CG methylation even why being activated by the HiTG transgene. This CG methylation is likely why it snaps back to silenced when the HiTG transgene is segregated away. But why do you think the Hi element is activated in the first place when it retains some CG (panel C)? Is there a threshold of CG that needs to be removed to be activated? Is Hi partially activated, and that is why it can snap back to silenced? While other elements that lose ALL CG are not able to be resilienced?

Minor changes to be made
1. Ddm1 and rts1 mutants are brought up in the Results section without an introduction. Please add a sentence defining what these plants are mutant for and why they have TE activation.
2. The numbers in the Results section "Targets of RdDM and VANC overlap" do not require >10 significant digits and should be shortened.
3. In the Discussion, McClintock is referenced in a sentence that refers to DNA methylation. McClintock did not investigate methylation, and another reference should be added to this sentence to make this clear.
4. Figure 4C - the colors are too light and smashed together with other colors to see them. Perhaps label each line, or somehow make the different genotypes more easy to discern.

Referee #2:

This manuscript provides evidence that re-silencing of previously reactivated Vandal elements requires various components of the RdDM pathway. The experiments are well designed and the conclusions supported. However, I had some difficulty understanding the logic of the interpretations and would appreciate a careful explanation of points that I found questionable or

difficult to understand. The biggest issue is the source of information used for re-silencing. Is it residual methylation in the elements themselves? I assume so, since removal of all CG methylation was sufficient to prevent re-silencing in wt progenies of met1 mutants. Is this the same or different from other instance in which silencing is heritably triggered?

"Sequence-specific anti-silencing allows these TEs to proliferate with minimum host damage." How so? They protect the TE from silencing. How does that protect the host? Because it is better than a global system of silencing reversal?

"should occasionally be active" I would say are, or have been, instead of should.

"remain mobile even in the wild-type" that is true in that case, but it isn't always the case, is it?

"found in TEs with small RNA" with small RNAs that target them?

"homology-based silencing is mediated by small RNA." Yes, but this might be an opportunity to emphasize that not all small RNAS are trans-acting, and that the "de-novo" methylation associated with classical RdDM refers to de-novo methylation of already methylated sequences, not necessarily de-novo trans silencing.

"proteins would counteract" don't need the word "would"

"This specific anti-silencing would allow the VANDAL TEs to proliferate with minimum damage to the host" You have mentioned this more than once, but you haven't explained by this process has anything to do with minimizing damage to the host.

Figure 1. Perhaps include the ends of the element in 1b?
F1c: details are fine in the M&M, but at least include units in the figure itself. Similarly, explain what red and black mean here.
"(hereafter called cmt23), Hi was re-silenced when HiTG segregated" For clarity, maybe segregated away? Or in plants in which HiTG was lost via segregation?

"Hi often remained mobile even after the loss of HiTG (Fig. 2B)." The often part is interesting. Is there anything else segregating in these families? As, I see you have noted. It would be interesting of you looked at larger numbers to see if that other something is segregating as a locus.

"Thus, ddcc mutation has a strong impact on the de novo immobilization of Hi, even though immobilized Hi remains immobile in the ddcc background." Very nice experiment.

"Hi was also mobilized in ddm1 and rts1 mutants, which also abolished mCG in TEs" make clear in the text that this is independent of past exposure to HiTG. The initiation/maintenance distinction is often lost on readers unfamiliar with the field. I am also a fan of an upfront figure showing your crossing schemes.

"after the loss of HiTG, drm12" should be the drm12 mutations

"DRM activity affected recovery around the" for clarity, "DRM activity affected recovery of methylation around the"

"Under such conditions, Hi was completely silenced and immobilized" I would have liked to see the TSSs for each of the genes in the cartoons of the TEs.

"The results above demonstrate that RdDM targets noncoding regions, as is the case for anti-silencing by VANC21." Are these non-coding regions transcribed or not?

"Taken together, these results indicate that RdDM and VANC regulate common target regions in opposite directions." I would have liked to see a zoom in of the methylation status of the repeat sequences that are targeted by VANC (and presumably RdDM). Interesting that selection has favored the presence of tandem repeats in the TEs, given that tandems are often targets of silencing. I wonder if that propensity is being used by VANC to specifically recognizing those sequences.

"when HiTG is segregated." Segregated away?

"HiTG, while mCG remained in activated" While should be start of new sentence.

"makes Hi tolerant to silencing by RdDM," I would say prone to, or subject to.

"These results indicate that complete loss of mCG makes Hi tolerant to silencing by RdDM." So this suggests that methylation of the target TE is required for triggering of methylation by siRNAs?

"of body mCH in TE silencing remains to be explored" or in genes.

"would lead to their long-term advantage to maintain host fitness." Again, I'm not clear where this assertion is coming from.

"It is tempting to speculate that the short-term advantage of differentiation of the VANC systems for the TEs s the escape from RNAi." This is getting a little murky for me, I suspect that many of these issues have been discussed in previous papers. I think what is throwing me is the trigger for silencing vai RdDM. Is it small already methylated elements, so if an element is making a little bit of smRNAs, it is sufficient to reestablish full methylation? Or is it small RNAs from other elements in the background? If it is from the elements themselves, then having the targets of methylation diverge wouldn't make sense, because they are carrying the seeds of their own silencing. If not, then what is silencing them? Perhaps this could be discussed more fully.

"An Spm-encoded protein called TnpA has been shown to activate Spm, which correlates with DNA demethylation (Schläppi et al. 1994; Cui & Fedoroff 2002)." However, although MURA demethylates sequences in the TIRs of non-autonomous elements, it does not heritably reverse that methylation, nor does it heritably reactivate previously silenced MuDR elements.

Referee #3:

This manuscript follows up on previous work from the Kakutani laboratory that identified the VANC anti-silencing system in which VANC proteins encoded by some transposable elements of the VANDAL family can induce loss of DNA methylation and mobilization of related elements.
In this manuscript, Sasaki et al focus of the VANDAL21 elements and address the question of whether and how activated elements can be re-silenced. Through genetic approaches using a transgene containing a full-length copy of the VANDAL21 Hi element to activate the endogenous Hi copy and several mutants defective for RNA-directed DNA methylation (RdDM), the authors show that Hi is resilenced and remethylated in an RdDM-dependent manner following segregation of the transgene, and that CG methylation is crucial to Hi silencing. CMT2 and CMT3 are also involved in Hi resilencing in the absence of the typical RdDM effector DRM2.
Interestingly, RdDM-mediated resilencing preferentially targets non-coding regions of Hi, which also happen to be the regions bound by the VANC21 protein, indicating that anti-silencing and resilencing systems target common regions. Finally, the authors show that complete loss of CG methylation at Hi precludes its re-silencing by RdDM, and that non-CG methylation in coding regions of VANDAL elements is required for transcriptional repression.

That resilencing/remethylation of VANDAL21 requires DRMs and a certain level of CG methylation has been shown for other TEs and is not unexpected. However, the finding that both resilencing and anti-silencing mechanisms target common TE regions provide interesting new insights into the arms race between silencing and anti-silencing and would be of broad interest to TE community as well as to evolutionary biologists. The manuscript is clearly written and easy to read, the experiments are well designed, and the data sound.

I have only minor comments/suggestions:
- The authors (rightly) use excision of Hi as a proxy for its activity. I think it would be informative to show the corresponding transcription data, for instance to exclude the possibility that potential Hi neo-copies might be active in backgrounds where excision of Hi is not detected, or in case excision and transcription might be uncoupled.
- RdDM-mediated resilencing is expected to involve production of siRNAs corresponding to the targeted regions. It would be informative to determine whether siRNA originating from Hi accumulate (in particular in its non-coding, VANC target regions) and if so, whether production of siRNAs is influenced by DNA methylation of the corresponding regions.
- The rational for using regions that loose mCG as target regions of VANC21 instead of VANC21 peaks extracted from available VANC21 ChIP-seq data is unclear.
- The authors use the term "RNAi" throughout the manuscript. In the introduction, they distinguish RNAi from DNA methylation and histone modifications as epigenetic mechanisms that silence TEs, suggesting that the "RNAi" is used here to refer to silencing mechanisms acting at the post-transcriptional. As the manuscript focuses on the involvement of RdDM in VANDAL resilencing, I would suggest using RdDM instead of RNAi.
- I found the word "tolerant" in the sentence "These results indicate that complete loss of mCG makes Hi tolerant to silencing by RdDM" possibly ambiguous. I would suggest changing it for insensitive or refractory.

We thank the editor and all the reviewers for their constructive comments. We incorporated most of the points, which we believe has improved the manuscript significantly.

Referee #1:

This is a review of the manuscript entitled "The arms race in noncoding regions of transposable elements: the evolution of ant-silencing and RNAi" submitted to EMBO Reports by Sasaki et al.
This work uses both a transgene system and mutant backgrounds to study the activation and resilencing of endogenous VANDAL transposable elements in Arabidopsis. The strength of the work is the ability to mechanistically separate the roles of CG DNA methylation and RNA-directed DNA methylation (RdDM), as well as the analysis of the co-regulation of particular non-coding regions of VANDAL elements by both RdDM and VANDAL-encoded anti-silencing proteins.

We thank the Referee #1 for the positive evaluation.

Major issues
1. In both the title and abstract, the term "evolution" is used. For example, the abstract says "Target motifs of VANC, in turn, evolved to escape RNAi". However, there is no sequence analysis of evolution in this work, and therefore the authors should not make evolutionary arguments. They can certainly say that this region is co-regulated, but saying "evolved to.." would take different sequence analysis experiments that were not performed here.

As the Referee pointed out, the evolution of target motifs is not shown in this work; it has been shown in our previous publications (Sasaki et al 2022 EMBO J; Hosaka et al 2017 Nat Commun). In order to make this point clearer, we moved the description of the sequence divergence in introduction part (before “Here, we show…”) of the abstract, with modification of the expression to “Target regions of VANCs in the noncoding regions form tandem repeats, which diverge rapidly and synchronously”. We also modified other parts of the manuscript, including the title, to make this point clearer.

2. In the abstract and many times in the body, the authors refer to RNAi when they mean RdDM. This work did not test RDR6, SGS3 nor other RNAi proteins. It only tested RdDM proteins.

We agree with this point and changed the word “RNAi” to “RdDM ”throughout the manuscript.

3. The Results remind me of the SPM and Mutator systems with autonomous and non-autonomous elements. The non-autonomous elements are full methylated, but then in the presence of the transposase

protein from the active autonomous element, they become unmethylated, only to snap back to methylated when the autonomous element is segregated away. See the work of Damon Lisch using the Mutator system in maize. In this case, the methylated state can be thought of as the default, and only temporarily unmethylated in the presence of TE-binding protein. A similar phenomenon is likely happening here.

We agree that VANC system has similarity to other TE-mediated demethylation systems. We added discussion on other TE systems including those in Spm and MuDR in Line 306-316.

4. In the Results that describe Figure 2D, the authors describe that the TE is not mobilized in the ddcc mutant. Yet the data shows that the TE is not mobilized in any RdDM mutant. Please broaden the analysis and speculate what this means about the TE activation seen in the literature and field that occurs in any RdDM mutant. This is important because many researchers say that RdDM suppresses transposition, when actually there is very little evidence that it is important for transposition at all.

We agree with the comment about the role of RdDM for transposition. We added the following discussion about the effect of RdDM on TE regulation in Line 277.
"Compared to *met1* or *ddm1* mutant, which affect maintenance of mCG, loss of RdDM function has only a minor impact on the TE derepression within the Arabidopsis genome (Huettel et al 2006; Ito et al. 2011; Baduel et al 2021). Consistent with this general trend, *Hi* is not activated in RdDM mutant, but RdDM has significant importance for *de novo* silencing of *Hi* (Fig.2; Appendix Fig S2)."

5. In the Results section "Targets of RdDM and VANC overlap", this overlap analysis of two datasets needs negative controls such as random regions of the genome or VANC regions. Pie charts is not the best way to show the overlap in this data. An analysis of observed overlap in these datasets compared to what would be expected by chance with datasets this size is more appropriate.

As suggested, we added Venn diagrams for randomly chosen regions (Appendix Figure S8).

6. The last Results section is poorly written. It is unclear what this all means. Please write this section with effort to guide the reader of what these results mean.

We changed the way to write the last results section to guide readers to follow the meaning of the results. We try to put the results in the context of VANC functions for the removal of mCH in the coding regions of VANDALs. We believe this change has made the manuscript easier to understand for the readers. We thank the Referee #1 for the suggestion.

7. In this same last Results section, the authors claim that the body of the VANC element is regulated differently. To make this claim, perform an analysis that compares the body, ends, coding, non-coding, etc... regions.

We modified Figure 6B to add results for DNA methylation status in divided regions, such as coding and noncoding, and within noncoding regions, TIR, intergenic, and introns.

8. In Figure 3, it would help to add the methylation pattern of ddcc as a reference to compare drm12 to. Is all VANC methylation absent in ddcc?

We added metaplot data of DNA methylation over VANDAL TE in *ddcc* mutant background in Appendix Figure S7. All mCH are removed in *ddcc*, but mCG remains in non-VANC target regions. This data also supports the idea that mCG and mCH have different roles in VANC-mediated anti-silencing, and clearly shows the CG-methylated regions correlating with the initiation of activation.

9. Throughout the entire manuscript, it is clear that CG methylation controls VANC element expression. However, in Figure S6, the endogenous Hi element retains some CG methylation even why being activated by the HiTG transgene. This CG methylation is likely why it snaps back to silenced when the HiTG transgene is segregated away. But why do you think the Hi element is activated in the first place when it retains some CG (panel C)? Is there a threshold of CG that needs to be removed to be activated? Is Hi partially activated, and that is why it can snap back to silenced? While other elements that lose ALL CG are not able to be resilienced?

As the Referee pointed out, $\mathrm{Hi}^{\mathit{Hi}\mathrm{TG}}$ is CG methylated, but RT-PCR showed that endogenous VANC21 is expressed (Appendix Fig S1B), so we considered endogenous *Hi* was activated even in the presence of mCG. We think specific region hypomethylated by VANC is important for the activation of encoded genes. As a response to Referee #1’s major comment 8, we added Appendix Figure S7 that shows DNA methylation status of VANDAL21 in *ddcc* (immobile) and *ddcc* (-*Hi*TG) (mobile), and it supports the view that mCG in noncoding regions are critical for the initiation of TE activation.

Minor changes to be made

1. Ddm1 and rts1 mutants are brought up in the Results section without an introduction. Please add a sentence defining what these plants are mutant for and why they have TE activation.

We added explanations for *DDM1* in the Introduction part. We also added an explanation of *RTS1* in the legend of Fig 2D in Line 681.

2. The numbers in the Results section "Targets of RdDM and VANC overlap" do not require >10 significant digits and should be shortened.

We agree with this point and shortened the values in the main text (Lines 193-196) and figure legend (Line 699). We also noticed that the numbers in hypergeometric test for VANC1 target were wrong and corrected that (shown in red in Lines 195, 196, and 699).

3. In the Discussion, McClintock is referenced in a sentence that refers to DNA methylation. McClintock did not investigate methylation, and another reference should be added to this sentence to make this clear.

As the Referee #1 pointed out, she did not study DNA methylation, and later works by others showed the effect on DNA methylation. We changed sentences to clarify her discovery and later studies on Spm in Line 307.

4. Figure 4C - the colors are too light and smashed together with other colors to see them. Perhaps label each line, or somehow make the different genotypes more easy to discern.

We changed the colors to make it easy to discern.

Referee #2:

This manuscript provides evidence that re-silencing of previously reactivated Vandal elements requires various components of the RdDM pathway. The experiments are well designed and the conclusions supported.

We thank the Referee #2 for the positive evaluation.

However, I had some difficulty understanding the logic of the interpretations and would appreciate a careful explanation of points that I found questionable or difficult to understand. The biggest issue is the source of information used for re-silencing. Is it residual methylation in the elements themselves? I assume so, since removal of all CG methylation was sufficient to prevent re-silencing in wt progenies of met1 mutants. Is this the same or different from other instance in which silencing is heritably triggered?

As the Referee #2 assumed, we are considering that complete loss of mCG has a strong impact for stable inheritance of TE derepression. Stable inheritance of TE derepression is known genome-wide for

outcrossed progeny from *ddm1* or *met1*; in both instances, the stable inheritance is associated with loss of mCG (Saze et al 2003; Kato et al 2004; Quadraba et al 2019). Strong impact of mCG can be understood through multiple positive feedback operating for specific silencing of TEs (To & Kakutani 2022 Curr Opin; To et al 2022 Nat Commun; Choi et al 2021 Elife). We modified related discussion in Lines 299-305.

"Sequence-specific anti-silencing allows these TEs to proliferate with minimum host damage." How so? They protect the TE from silencing. How does that protect the host? Because it is better than a global system of silencing reversal?

Global derepression of TEs is expected to have severe damage to the host, due to mutagenic effects, perturbation of nearby host genes, and ectopic recombination. Compared to that, damage to the host is expected to be minimal for the sequence-specific anti-silencing. We modified this part to make the logic clearer. We added discussion that sequence-specific anti-silencing is less deleterious than the global anti-silencing in comparison to the anti-defense in viruses (Line 97). We thank the Referee #2 for asking those questions, as that certainly improves the manuscript.

"should occasionally be active" I would say are, or have been, instead of should.

As suggested, we changed the expression to “TEs have been occasionally active” in Line 34.

"remain mobile even in the wild-type" that is true in that case, but it isn't always the case, is it?

As the Referee #2 pointed out, some TEs keep activities in WT background, but some are silenced in later generations. We specified the TEs in Line 59.

"found in TEs with small RNA" with small RNAs that target them?

We changed this part as suggested in Line 63.

"homology-based silencing is mediated by small RNA." Yes, but this might be an opportunity to emphasize that not all small RNAS are trans-acting, and that the "de-novo" methylation associated with classical RdDM refers to de-novo methylation of already methylated sequences, not necessarily de-novo trans silencing.

In order to include multiple pathways of RdDM, we added citations of Cuerda-Gil & Slotkin (2016), Fultz & Slotkin (2017), and Sigman et al. (2021) in Line 66. We would like to refrain from explaining their details, because that is not directly related to our results. We added the introduction of DRMs here.

"proteins would counteract" don't need the word "would"

As suggested, we removed “would” from sentence in line 83.

"This specific anti-silencing would allow the VANDAL TEs to proliferate with minimum damage to the host" You have mentioned this more than once, but you haven't explained by this process has anything to do with minimizing damage to the host.

As mentioned above, added explanation in Line 97.

Figure 1. Perhaps include the ends of the element in 1b?
F1c: details are fine in the M&M, but at least include units in the figure itself. Similarly, explain what red and black mean here.

In Fig 1b, the ends of the element are shown by dashed lines. We added the explanation in the legend of this Figure for clarity. Figure 1C shows non-dimensional values reflecting significance of changes in DNA methylation.

"(hereafter called cmt23), Hi was re-silenced when HiTG segregated" For clarity, maybe segregated away? Or in plants in which HiTG was lost via segregation?

As suggested, we changed this expression to “segregated away” in Line 136 and others with same expression.

"Hi often remained mobile even after the loss of HiTG (Fig. 2B)." The often part is interesting. Is there anything else segregating in these families? As, I see you have noted. It would be interesting of you looked at larger numbers to see if that other something is segregating as a locus.

We thank the Referee #2 for asking the very interesting question. We still do not know if the mobilization is a purely stochastic event or depends on segregating trans-acting loci. We do not have any evidence for the latter possibility so far. Although *Hi* is the major mobile and autonomous copy of *VANDAL21* mobilized in the *ddm1* mutant (Tsukahara et al 2009, Fu et al 2013), it is still possible that methylation status of related copies in the other loci modify the *Hi* mobility. While that would certainly be an interesting question for future research, we would like to refrain from discussing that in this manuscript.

"Thus, ddcc mutation has a strong impact on the de novo immobilization of Hi, even though immobilized Hi remains immobile in the ddcc background." Very nice experiment.

We appreciate this comment by Referee #2.

"Hi was also mobilized in ddm1 and rts1 mutants, which also abolished mCG in TEs" make clear in the text that this is independent of past exposure to HiTG. The initiation/maintenance distinction is often lost on readers unfamiliar with the field. I am also a fan of an upfront figure showing your crossing schemes.

We clarified this point by adding "even without previous exposure to *Hi*TG" within this paragraph when we explained the effect of *met1* mutation (Line 159). In the last sentence of this paragraph, we also added the explanation that mCG is important for maintenance of VANDAL21 immobilization (Line 162).

"after the loss of HiTG, drm12" should be the drm12 mutations

As suggested, we changed this expression to "after the loss of *Hi*TG, *drm12* mutations … " in Line 172.

"DRM activity affected recovery around the" for clarity, "DRM activity affected recovery of methylation around the"

We changed this part as suggested in Line 175.

"Under such conditions, Hi was completely silenced and immobilized" I would have liked to see the TSSs for each of the genes in the cartoons of the TEs.

This is an important but difficult question. Although TSS of VANB is within VANDAL TEs, RNA-seq in *ddm1* showed TSSs of VANA and VANC are outside of TEs. This is probably consistent with the fact that *Hiun* is preferentially inserted upstream of genes (Fu et al. 2013), and newly inserted copies may use original TSSs of genes flanking to insertion points. In case of VANC21 of *Hiun*, TSS is outside and it is not methylated when *Hi* is re-silenced. It is clear that when *Hi* is re-silenced, VANDAL TEs restore DNA methylation completely, but how TSS sites are regulated is still elusive. We believe our experimental data would be significantly strong to show the effect of DNA re-methylation for re-silencing of *Hi*, and we will try to answer this point by further experiments in the future.

"The results above demonstrate that RdDM targets noncoding regions, as is the case for anti-silencing by VANC21." Are these non-coding regions transcribed or not?

RNA-seq in *ddm1* mutant (Oberlin et al. 2017) showed that intronic regions of VANDAL-encoded genes are spliced out, and TIRs and intergenic regions (regions between VANA and VANB, and VANB and VANC) do not generate RNA.

"Taken together, these results indicate that RdDM and VANC regulate common target regions in opposite directions." I would have liked to see a zoom in of the methylation status of the repeat sequences that are targeted by VANC (and presumably RdDM). Interesting that selection has favored the presence of tandem repeats in the TEs, given that tandems are often targets of silencing. I wonder if that propensity is being used by VANC to specifically recognizing those sequences.

We added zoom-up picture of DNA methylation status in the motif-rich regions below. mCHH by RdDM is induced near these regions, but the overlap is not perfect. Similarly, siRNA-generating regions are not always matched with motif-rich regions, as we showed in the newly added Appendix Fig S3. Interaction among tandem repeats, siRNA, and DNA methylation would be interesting subjects for future study.

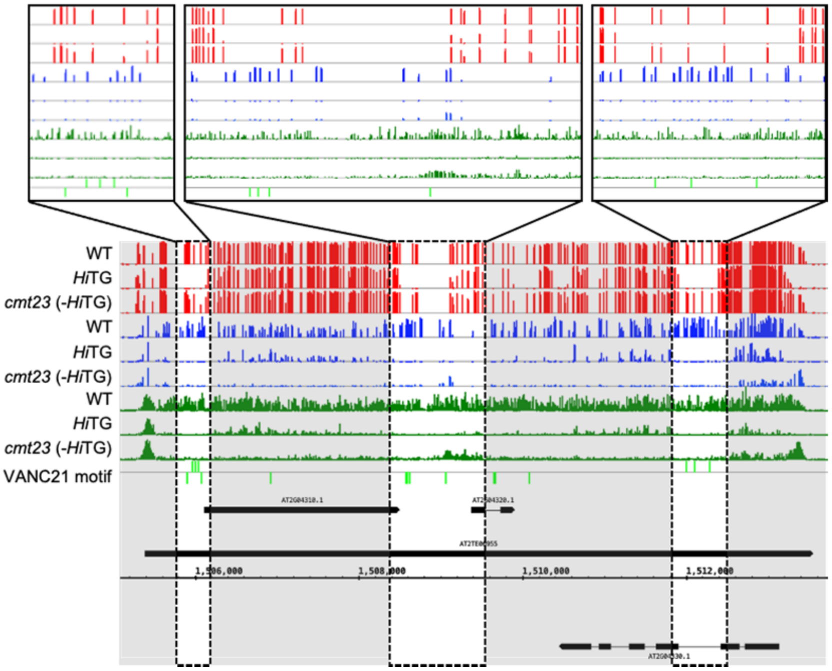

"when HiTG is segregated." Segregated away?

We changed this expression to “when *Hi*TG is segregated away.”.

"HiTG, while mCG remained in activated" While should be start of new sentence.

We changed this sentence to “*Hi*TG; while mCG remained in activated …” in Line 212.

"makes Hi tolerant to silencing by RdDM," I would say prone to, or subject to.

We changed this expression to “makes Hi refractory to silencing by RdDM” in Line 229.

"These results indicate that complete loss of mCG makes Hi tolerant to silencing by RdDM." So this suggests that methylation of the target TE is required for triggering of methylation by siRNAs?

We thank the important point by Referee #2. There would be two possibilities for the role of mCG for re-silencing through RdDM; [1] mCG is required for triggering DNA methylation, and [2] mCG is required for generation of siRNA, namely, for recruitment of RdDM machineries to target. Now we don’t have a conclusive answer yet, so we added these possibilities in Line 299.

"of body mCH in TE silencing remains to be explored" or in genes.

We removed this part.

"would lead to their long-term advantage to maintain host fitness." Again, I'm not clear where this assertion is coming from.

We added explanations in Lines 97 and 272.

"It is tempting to speculate that the short-term advantage of differentiation of the VANC systems for the TEs s the escape from RNAi." This is getting a little murky for me, I suspect that many of these issues have been discussed in previous papers. I think what is throwing me is the trigger for silencing vai RdDM. Is it small already methylated elements, so if an element is making a little bit of smRNAs, it is sufficient to reestablish full methylation? Or is it small RNAs from other elements in the background? If it is from the elements themselves, then having the targets of methylation diverge wouldn't make sense, because they are carrying the seeds of their own silencing. If not, then what is silencing them? Perhaps this could be discussed more fully.

We added the following explanation to make this point clearer in Line 272.

“For example, although VANDAL21 and VANDAL6 are similar in their overall sequences, they are different in sequences where VANC21 and VANC6 proteins affect (Hosaka et al 2017), presumably enabling escape of VANDAL21 copies from RdDM targeting to VANDAL6 copies.”

"An Spm-encoded protein called TnpA has been shown to activate Spm, which correlates with DNA demethylation (Schläppi et al. 1994; Cui & Fedoroff 2002)." However, although MURA demethylates sequences in the TIRs of non-autonomous elements, it does not heritably reverse that methylation, nor does it heritably reactivate previously silenced MuDR elements.

As the Referee #2 suggested, a transposase of MuDR TE, demethylates TIR regions of MuDR TEs, but they are easily remethylated when MURA is segregated away (Burgess et al. 2020). We added the citation of this interesting work and discussed the similarity in Line 310.

Referee #3:

This manuscript follows up on previous work from the Kakutani laboratory that identified the VANC anti-silencing system in which VANC proteins encoded by some transposable elements of the VANDAL family can induce loss of DNA methylation and mobilization of related elements.
In this manuscript, Sasaki et al focus of the VANDAL21 elements and address the question of whether and how activated elements can be re-silenced. Through genetic approaches using a transgene containing a full-length copy of the VANDAL21 Hi element to activate the endogenous Hi copy and several mutants defective for RNA-directed DNA methylation (RdDM), the authors show that Hi is resilenced and remethylated in an RdDM-dependent manner following segregation of the transgene, and that CG methylation is crucial to Hi silencing. CMT2 and CMT3 are also involved in Hi resilencing in the absence of the typical RdDM effector DRM2.
Interestingly, RdDM-mediated resilencing preferentially targets non-coding regions of Hi, which also happen to be the regions bound by the VANC21 protein, indicating that anti-silencing and resilencing systems target common regions. Finally, the authors show that complete loss of CG methylation at Hi precludes its re-silencing by RdDM, and that non-CG methylation in coding regions of VANDAL elements is required for transcriptional repression.

1st Revision - Editorial Decision 7th May 2023

Dear Dr. Sasaki,

Thank you for the submission of your revised manuscript. We have now received the enclosed reports from the referees and I am happy to say that all support its publication now. Only a few editorial requests will need to be addressed before we can proceed with the official acceptance of your manuscript.

- Please add up to 5 keywords to the ms file.

- Please rename the COI subheading to "Disclosure and Competing Interest Statement"

- Please remove the author contributions from the ms file. We now use CRediT to specify the contributions of each author in the journal submission system. CRediT replaces the author contribution section. Please use the free text box to provide more detailed descriptions, if you wish. See also guide to authors https://www.embopress.org/page/journal/14693178/authorguide#authorshipguidelines.

- Regarding the REFERENCE FORMAT, please correct to "et al" after 10 author names.

- The FIGURE CALLOUTS for Figure 5A and Figure 5B are missing, please add.

- Please remove the Appendix figure/table legends from the main ms file and add them to the Appendix file.

- The source data should be uploaded as one file per figure. Please zip all source data of one figure together and upload the zipped file.

- I attach to this email a related ms file with comments by our data editors. Please address all comments in the final ms.

EMBO press papers are accompanied online by A) a short (1-2 sentences) summary of the findings and their significance, B) 2-3 bullet points highlighting key results and C) a synopsis image that is exactly 550 pixels wide and 200-600 pixels high (the height is variable). You can either show a model or key data in the synopsis image. Please note that text needs to be readable at the final size. Please send us this information along with the final manuscript.

I would like to suggest some minor changes to the title and abstract. Please let me know whether you agree with the following:

Arms race in noncoding regions of transposable elements between anti-silencing and RdDM

Transposable elements (TEs) are among the most dynamic parts of genomes. Since TEs are potentially deleterious, eukaryotes silence them through epigenetic mechanisms such as repressive histone modifications and DNA methylation. We previously reported that Arabidopsis TEs, called VANDALs, counteract epigenetic silencing through a group of sequence-specific anti-silencing proteins, VANCs. VANC proteins bind to noncoding regions of specific VANDAL copies and induce loss of silent chromatin marks. The VANC target regions form tandem repeats, which diverge rapidly and synchronously [what do you mean by synchronously? Synchronous with what? Please explain or delete]. Sequence-specific anti-silencing allows these TEs to proliferate with minimum host damage. Here, we show that RNA-directed DNA methylation (RdDM) efficiently targets noncoding regions of VANDAL TEs to silence them de novo. Thus, escape from RdDM could be a primary event leading to the rapid evolution and diversification [OK?] of sequence-specific anti-silencing systems. We propose that this selfish behavior of TEs paradoxically could make them diverse and less harmful to the host.

I look forward to seeing a final version of your manuscript as soon as possible. Please use this link to submit your revision: https://embor.msubmit.net/cgi-bin/main.plex

Best regards,
Esther

Esther Schnapp, PhD
Senior Editor
EMBO reports

Referee #1:

The authors did a very good job responding to my review and improving the manuscript.

Referee #2:

The revisions have satisfied my concerns. Reviewing manuscripts by members of this research group is always a pleasure.

Referee #3:

The authors satisfactorily addressed my previous points. I have no further comment on the revised manuscript.

The authors have addressed all minor editorial requests.

2nd Revision - Editorial Decision **19th May 2023**

Dr. Taku Sasaki
The University of Tokyo
Department of Biological Sciences
Japan

Dear Dr. Sasaki,

I am very pleased to accept your manuscript for publication in the next available issue of EMBO reports. Thank you for your contribution to our journal.

At the end of this email I include important information about how to proceed. Please ensure that you take the time to read the information and complete and return the necessary forms to allow us to publish your manuscript as quickly as possible.

As part of the EMBO publication's Transparent Editorial Process, EMBO reports publishes online a Review Process File to accompany accepted manuscripts. As you are aware, this File will be published in conjunction with your paper and will include the referee reports, your point-by-point response and all pertinent correspondence relating to the manuscript.

If you do NOT want this File to be published, please inform the editorial office within 2 days, if you have not done so already, otherwise the File will be published by default [contact: emboreports@embo.org]. If you do opt out, the Review Process File link will point to the following statement: "No Review Process File is available with this article, as the authors have chosen not to make the review process public in this case."

Thank you again for your contribution to EMBO reports and congratulations on a successful publication. Please consider us again in the future for your most exciting work.

Yours sincerely,

Esther Schnapp, PhD
Senior Editor
EMBO reports

****

THINGS TO DO NOW:

Please note that you will be contacted by Wiley Author Services to complete licensing and payment information. The required 'Page Charges Authorization Form' is available here: https://www.embopress.org/pb-assets/embo-site/er_apc.pdf - please download and complete the form and return to embopressproduction@wiley.com

EMBO Press participates in many Publish and Read agreements that allow authors to publish Open Access with reduced/no publication charges. Check your eligibility: https://authorservices.wiley.com/author-resources/Journal-Authors/open-access/affiliation-policies-payments/index.html

You will receive proofs by e-mail approximately 2-3 weeks after all relevant files have been sent to our Production Office; you should return your corrections within 2 days of receiving the proofs.

Please inform us if there is likely to be any difficulty in reaching you at the above address at that time. Failure to meet our deadlines may result in a delay of publication, or publication without your corrections.

All further communications concerning your paper should quote reference number EMBOR-2022-56678V3 and be addressed to emboreports@wiley.com.

Should you be planning a Press Release on your article, please get in contact with emboreports@wiley.com as early as possible, in order to coordinate publication and release dates.

EMBO Press Author Checklist

Corresponding Author Name: Taku Sasaki	
Journal Submitted to: The EMBO Reports	
Manuscript Number: EMBOR-2022-56678V2	

USEFUL LINKS FOR COMPLETING THIS FORM
The EMBO Journal - Author Guidelines
EMBO Reports - Author Guidelines
Molecular Systems Biology - Author Guidelines
EMBO Molecular Medicine - Author Guidelines

Reporting Checklist for Life Science Articles (updated January 2022)
This checklist is adapted from Materials Design Analysis Reporting (MDAR) Checklist for Authors. MDAR establishes a minimum set of requirements in transparent reporting in the life sciences (see Statement of Task: 10.31222/osf.io/9sm4x). Please follow the journal's guidelines in preparing your manuscript.
Please note that a copy of this checklist will be published alongside your article.

Abridged guidelines for figures
1. Data
The data shown in figures should satisfy the following conditions:
- the data were obtained and processed according to the field's best practice and are presented to reflect the results of the experiments in an accurate and unbiased manner.
- ideally, figure panels should include only measurements that are directly comparable to each other and obtained with the same assay.
- plots include clearly labeled error bars for independent experiments and sample sizes. Unless justified, error bars should not be shown for technical replicates.
- if n<5, the individual data points from each experiment should be plotted. Any statistical test employed should be justified.
- Source Data should be included to report the data underlying figures according to the guidelines set out in the authorship guidelines on Data Presentation.

2. Captions
Each figure caption should contain the following information, for each panel where they are relevant:
- a specification of the experimental system investigated (eg cell line, species name).
- the assay(s) and method(s) used to carry out the reported observations and measurements.
- an explicit mention of the biological and chemical entity(ies) that are being measured.
- an explicit mention of the biological and chemical entity(ies) that are altered/varied/perturbed in a controlled manner.
- the exact sample size (n) for each experimental group/condition, given as a number, not a range;
- a description of the sample collection allowing the reader to understand whether the samples represent technical or biological replicates (including how many animals, litters, cultures, etc.).
- a statement of how many times the experiment shown was independently replicated in the laboratory.
- definitions of statistical methods and measures:
  - common tests, such as t-test (please specify whether paired vs. unpaired), simple χ2 tests, Wilcoxon and Mann-Whitney tests, can be unambiguously identified by name only, but more complex techniques should be described in the methods section;
  - are tests one-sided or two-sided?
  - are there adjustments for multiple comparisons?
  - exact statistical test results, e.g., P values = x but not P values < x;
  - definition of 'center values' as median or average;
  - definition of error bars as s.d. or s.e.m.

Please complete ALL of the questions below.
Select "Not Applicable" only when the requested information is not relevant for your study.

Materials

Newly Created Materials	Information included in the manuscript?	In which section is the information available? (Reagents and Tools Table, Materials and Methods, Figures, Data Availability Section)
New materials and reagents need to be available; do any restrictions apply?	Not Applicable	

Antibodies	Information included in the manuscript?	In which section is the information available? (Reagents and Tools Table, Materials and Methods, Figures, Data Availability Section)
For **antibodies** provide the following information:<br>- Commercial antibodies: RRID (if possible) or supplier name, catalogue number and or/clone number<br>- Non-commercial: RRID or citation	Not Applicable	

DNA and RNA sequences	Information included in the manuscript?	In which section is the information available? (Reagents and Tools Table, Materials and Methods, Figures, Data Availability Section)
**Short novel DNA or RNA including primers, probes**: provide the sequences.	Yes	Appendix Table S1

Cell materials	Information included in the manuscript?	In which section is the information available? (Reagents and Tools Table, Materials and Methods, Figures, Data Availability Section)
**Cell lines:** Provide species information, strain. Provide accession number in repository **OR** supplier name, catalog number, clone number, and/**OR** RRID.	Not Applicable	
**Primary cultures:** Provide species, strain, sex of origin, genetic modification status.	Not Applicable	
Report if the cell lines were recently **authenticated** (e.g., by STR profiling) and tested for mycoplasma contamination.	Not Applicable	

Experimental animals	Information included in the manuscript?	In which section is the information available? (Reagents and Tools Table, Materials and Methods, Figures, Data Availability Section)
**Laboratory animals or Model organisms:** Provide species, strain, sex, age, genetic modification status. Provide accession number in repository **OR** supplier name, catalog number, clone number, **OR** RRID.	Not Applicable	
**Animal observed in or captured from the field:** Provide species, sex, and age where possible.	Not Applicable	
Please detail **housing and husbandry conditions**.	Not Applicable	

Plants and microbes	Information included in the manuscript?	In which section is the information available? (Reagents and Tools Table, Materials and Methods, Figures, Data Availability Section)
**Plants:** provide species and strain, ecotype and cultivar where relevant, unique accession number if available, and source (including location for collected wild specimens).	Yes	Materials and Methods
**Microbes:** provide species and strain, unique accession number if available, and source.	Not Applicable	

Human research participants	Information included in the manuscript?	In which section is the information available? (Reagents and Tools Table, Materials and Methods, Figures, Data Availability Section)
If collected and within the bounds of privacy constraints report on age, sex and gender or ethnicity for all study participants.	Not Applicable	

Core facilities	Information included in the manuscript?	In which section is the information available? (Reagents and Tools Table, Materials and Methods, Figures, Data Availability Section)
If your work benefited from core facilities, was their service mentioned in the acknowledgments section?	Yes	Acknowledgments

Design

Study protocol	Information included in the manuscript?	In which section is the information available? (Reagents and Tools Table, Materials and Methods, Figures, Data Availability Section)
If study protocol has been **pre-registered, provide DOI in the manuscript**. For clinical trials, provide the trial registration number **OR** cite DOI.	Not Applicable	
Report the **clinical trial registration number** (at ClinicalTrials.gov or equivalent), where applicable.	Not Applicable	

Laboratory protocol	Information included in the manuscript?	In which section is the information available? (Reagents and Tools Table, Materials and Methods, Figures, Data Availability Section)
Provide DOI OR other citation details if **external detailed step-by-step protocols** are available.	Not Applicable	

Experimental study design and statistics	Information included in the manuscript?	In which section is the information available? (Reagents and Tools Table, Materials and Methods, Figures, Data Availability Section)
Include a statement about **sample size** estimate even if no statistical methods were used.	Yes	Figure, Figure Legends
Were any steps taken to minimize the effects of subjective bias when allocating animals/samples to treatment (e.g. **randomization procedure**)? If yes, have they been described?	Yes	Figure Legends
Include a statement about **blinding** even if no blinding was done.	Not Applicable	
Describe **inclusion/exclusion criteria** if samples or animals were excluded from the analysis. Were the criteria pre-established?<br><br>If sample or data points were omitted from analysis, report if this was due to attrition or intentional exclusion and provide justification.	Yes	Materials and Methods
For every figure, are **statistical tests** justified as appropriate? Do the data meet the assumptions of the tests (e.g., normal distribution)? Describe any methods used to assess it. Is there an estimate of variation within each group of data? Is the variance similar between the groups that are being statistically compared?	Yes	Materials and Methods

Sample definition and in-laboratory replication	Information included in the manuscript?	In which section is the information available? (Reagents and Tools Table, Materials and Methods, Figures, Data Availability Section)
In the figure legends: state number of times the experiment was **replicated** in laboratory.	Yes	Figure Legends
In the figure legends: define whether data describe **technical or biological replicates**.	Yes	Figure Legends

Ethics

Ethics	Information included in the manuscript?	In which section is the information available? (Reagents and Tools Table, Materials and Methods, Figures, Data Availability Section)
Studies involving **human participants**: State details of **authority granting ethics approval** (IRB or equivalent committee(s), provide reference number for approval.	Not Applicable	
Studies involving **human participants**: Include a statement confirming that **informed consent** was obtained from all subjects and that the experiments conformed to the principles set out in the WMA Declaration of Helsinki and the Department of Health and Human Services Belmont Report.	Not Applicable	
Studies involving **human participants:** For publication of **patient photos**, include a statement confirming that consent to publish was obtained.	Not Applicable	
Studies involving experimental **animals**: State details of **authority granting ethics approva**l (IRB or equivalent committee(s), provide reference number for approval. Include a statement of compliance with ethical regulations.	Not Applicable	
Studies involving **specimen and field samples:** State if relevant **permits** obtained, provide details of authority approving study; if none were required, explain why.	Not Applicable	

Dual Use Research of Concern (DURC)	Information included in the manuscript?	In which section is the information available? (Reagents and Tools Table, Materials and Methods, Figures, Data Availability Section)
Could your study fall under dual use research restrictions? Please check biosecurity documents and list of **select agents and toxins** (CDC): https://www.selectagents.gov/sat/list.htm	Not Applicable	
If you used a select agent, is the security level of the lab appropriate and reported in the manuscript?	Not Applicable	
If a study is subject to dual use research of concern regulations, is the name of the **authority granting approval and reference number** for the regulatory approval provided in the manuscript?	Not Applicable	

Reporting

The MDAR framework recommends adoption of discipline-specific guidelines, established and endorsed through community initiatives. Journals have their own policy about requiring specific guidelines and recommendations to complement MDAR.

Adherence to community standards	Information included in the manuscript?	In which section is the information available? (Reagents and Tools Table, Materials and Methods, Figures, Data Availability Section)
State if relevant guidelines or checklists (e.g., **ICMJE, MIBBI, ARRIVE, PRISMA**) have been followed or provided.	Not Applicable	
For **tumor marker prognostic studies,** we recommend that you follow the **REMARK** reporting guidelines (see link list at top right). See author guidelines, under 'Reporting Guidelines'. Please confirm you have followed these guidelines.	Not Applicable	
For **phase II and III randomized controlled trials**, please refer to the **CONSORT** flow diagram (see link list at top right) and submit the CONSORT checklist (see link list at top right) with your submission. See author guidelines, under 'Reporting Guidelines'. Please confirm you have submitted this list.	Not Applicable	

Data Availability

Data availability	Information included in the manuscript?	In which section is the information available? (Reagents and Tools Table, Materials and Methods, Figures, Data Availability Section)
Have **primary datasets** been deposited according to the journal's guidelines (see 'Data Deposition' section) and the respective accession numbers provided in the Data Availability Section?	Yes	Data Availability
Were **human clinical and genomic datasets** deposited in a public access-controlled repository in accordance to ethical obligations to the patients and to the applicable consent agreement?	Not Applicable	
Are **computational models** that are central and integral to a study available without restrictions in a machine-readable form? Were the relevant accession numbers or links provided?	Not Applicable	
If publicly available data were reused, provide the respective **data citations in the reference list**.	Yes	References
